# What happens after oil and gas decommissioning? A global systematic review of marine environmental effects

**DOI:** 10.1002/eap.70243

**Published:** 2026-04-23

**Authors:** Anaëlle J. Lemasson, Antony M. Knights

**Affiliations:** ^1^ School of Biological and Marine Sciences University of Plymouth Plymouth UK; ^2^ School of Biological, Earth and Environmental Sciences University College Cork Cork Ireland; ^3^ Environmental Research Institute University College Cork Cork Ireland

**Keywords:** abundance, artificial reefs, fish, marine invertebrates, oil rigs, OSPAR, policy

## Abstract

The thousands of oil and gas (OG) platforms placed at sea for fossil fuel extraction have introduced new hard substrate to the marine environment. Over time, these structures can become colonized by a diversity of marine life, fostering novel ecosystems. However, an increasing number of OG platforms are reaching decommissioning age and decisions regarding their fate must be made. Some view these artificial structures as litter that ought to be removed; others view them as valuable contributors to marine biodiversity worth preserving. Evidence of the environmental effects of these structures following different decommissioning strategies is needed to identify the potential benefits of each option and make informed decisions. Here, using a systematic synthesis approach, we show that our understanding of the effects of different decommissioning options is greatly limited by a lack of empirical evidence. Only three articles addressed the effects of OG removal, preventing firm conclusions either for or against this option. Most research focused on Rigs‐to‐Reefs options, revealing that reefed structures can create biodiverse systems, although with clear differences between reefing methods. Decommissioned structures with higher vertical relief (e.g., standing or topped) may offer higher ecological value than those with lower relief (e.g., toppled). Risks related to the decommissioning methods (e.g., harm from explosives, non‐native species introduction) are discussed. Despite the urgency, empirical research on decommissioning environmental impacts remains limited, particularly from the southern hemisphere. We call for coordinated international effort to establish standardization across decommissioning procedures, and monitoring and reporting requirements, to ensure that robust data are available to address this complex environmental challenge.

## INTRODUCTION

There is no question that the twin crises of climate change and biodiversity loss are some of the greatest challenges facing humanity today (Pörtner et al., 2023). The effects of contemporary trends in global warming on the environment and life on Earth are profound, with loss of biodiversity occurring at rates unseen since the Jurassic period. In response, many governments have set ambitious societal and environmental objectives as part of their commitment to reaching net‐zero by 2050 with a view to restrict global warming to 1.5°C above pre‐industrial levels (Rogelj et al., 2023). Decarbonization is central to this ambition. A rapid transition from our reliance on fossil fuels to alternative “green” energy sources is recognized as both necessary and urgent to meet increasing global energy demands while concomitantly reducing greenhouse gas emissions. In particular, offshore renewable (wind) energy (ORE) is increasingly promoted as an alternative energy source, with ambitious levels for global ORE production set out as 380 GW by 2030 and 2000 GW by 2050 (GWEC, 2022). This transition is anticipated to introduce tens of thousands of new structures into coastal and offshore areas already under ever‐increasing pressure from anthropogenic activities (Gourvenec et al., 2022; Gourvenec & Sykes, 2021).

Worldwide, thousands of aging offshore oil and gas (OG) platforms and their associated infrastructure that were installed for the exploration and production of fossil fuels are at or near end‐of‐life, and more will over time become redundant as part of the energy transition. The scale of decommissioning is daunting from a logistics, economic, and environmental standpoint; scientists, governments, industry, and policymakers are now realizing the considerable challenge that it presents. In some regions, such as the North Sea, offshore OG structures were designed large and heavy out of necessity to withstand the harsh environmental conditions structures are exposed to. For example, the concrete gravity base foundation Gullfaks C (North Sea) is the largest offshore structure in the world and weighs in excess of 1.5 m tons—its decommissioning represents an insurmountable challenge (Knights, Lemasson, Frost, & Somerfield, 2024). In the North Sea alone, at least approximately 2600 wells, approximately 1.2 million tons of topsides, and >130,000 tons of subsea infrastructure are scheduled for decommissioning over the next 6 years (OEUK, 2022). Perhaps ironically, the decommissioning challenges for OG will also apply to ORE, but the OG experience may grant hindsight and an opportunity to plan ahead as the sector continues to expand.

There is an ongoing debate over how to decommission these structures, and approaches to end‐of‐life management can vary greatly (see Bull & Love, 2019; Sommer et al., 2019). Mostly, options fall in one or more of the following categories: (i) complete removal of OG structures, whereby these are fully removed from the marine environment and brought onshore; (ii) various forms of reefing, usually of the subsurface jacket, such as “topping” (removal of upper section) or “toppling” where the jacket is laid horizontal on the seabed; or (iii) a combination of the two, which can be undertaken in situ or after relocation of the structure to a new site. A final category (iv) aims to repurpose structures to allow for other uses, such as for tourism, recreation, or renewable energy production.

International and regional regulations play an important role in end‐of‐life management by dictating which options may or may not be legally possible (see Jørgensen, 2012; Techera & Chandler, 2015; Trevisanut, 2020). International legal instruments (including the Geneva Convention on the Continental Shelf [1958], the United Nations Convention on the Law of the Sea [UNCLOS, 1982], and the guidelines of the International Maritime Organization [1989]) generally dictate that offshore structures must be fully removed from the marine environment to ensure navigation safety and environmental protection. Nevertheless, while regional and national regulations reinforce this stipulation (e.g., OSPAR Decision 98/3, the US Code of Federal Regulations), some also do allow for exemptions (also referred to as “derogations”). With no set international strategy, a dichotomous approach to decommissioning and derogations has been taken by different nations.

For some, they create “novel ecosystems” (Van Elden & Meeuwig, 2019) worthy of retention. For instance, in the United States, local derogations have allowed the repurposing of OG structures as artificial reefs (ARs) of more than 500 platforms since the 1980s, as part of their “Rigs‐to‐Reefs” (RtR) program (Kaiser & Shively, 2020). For others, structures are viewed as “litter” and where possible, should be removed. This is the case in the OSPAR area, where derogations to complete removal are seldom obtained despite the number of considerably larger and heavier structures there whose removal may not be feasible, compared to monopile foundations and steel jackets, the foundation of choice in the US Gulf of Mexico (GoM) and off the coast of California where RtR is widely implemented. Although more prominently known for its US popularity, RtR has been trialed or adopted in other regions for marine and coastal resources conservation, such as in Brunei Darussalam where a RtR policy exists since 1988, or more recently in Thailand. In Australia, while the default “base case” decommissioning option is full removal of infrastructure, the legislation allows other options—partial removal, remain in situ, and reefing—to be considered if shown (among other criteria) to provide equal or better environmental outcomes than the base case (Melbourne‐Thomas et al., 2021). The growing popularity of RtR around the world has led some scientists and nonscientists alike to suggest that the current policy of complete removal may not result in optimal environmental and societal outcomes (Knights, Lemasson, Firth, Beaumont, et al., 2024; Knights, Lemasson, Firth, Bond, et al., 2024; Sommer et al., 2019).

While acknowledging that environmental considerations are only one piece of the decommissioning puzzle, it is one that is often presented when in favor of leaving structures at sea. But does the scientific evidence on the ecological effects of alternative options such as RtR (or others) support their implementation? The need to make urgent international progress on decommissioning sparked a surge in discussions and research on the ecosystem effects of offshore structures, with several recent reviews attempting to collate and synthesize the available evidence base both regionally (Fowler et al., 2020; Melbourne‐Thomas et al., 2021) and globally (Fortune et al., 2024; Lemasson et al., 2022). It is largely accepted that the hard substrate and complexity provided by standing OG production platforms, that would otherwise be generally absent, provide an opportunity for colonization by epifaunal communities and habitat for fish that support a biodiverse local food web (reviewed in Fortune et al., 2024), creating novel ecosystems (Van Elden & Meeuwig, 2019). The environmental effects of standing platforms are not detailed here as they are reviewed in depth by Fortune et al. (2024). The question is whether this novel biodiversity associated with structures is beneficial and worthy of retention.

Ecological *benefit* can be defined as “positive or successful outcomes from species to habitat scales, such as enhanced biomass, diversity, and improved ecosystem functions” (Paxton et al., 2025). Often, the presence of OG structures is framed in a positive light (e.g., “acting as artificial reefs”), valuing the presence of added local biodiversity, abundance, or biomass irrespective of whether it is natural or novel, over the intrinsic value of unimpacted natural sedimentary areas. Whether biodiversity associated with structures is a true benefit (Schroeder & Love, 2004) or rather a “(hu)man‐made” construct that should be removed remains a contentious point of difference for stakeholders on either side of the debate. The notion of benefit or success is inherently subjective and differs with value systems, and local ecological, social, economic, and political objectives (Knights, Lemasson, Firth, Beaumont, et al., 2024; Knights, Lemasson, Firth, Bond, et al., 2024c). These perspectives alongside understanding the role standing production platforms play in the environment is unarguably important to informing their end‐of‐life management (Bull & Love, 2019; Sommer et al., 2019; Van Elden & Meeuwig, 2019), but importantly does not fully replace the need for direct empirical evidence of decommissioning effects.

Once decommissioned, a structure may not function as it did when it was still standing and active. Among other changes, resource extraction (and associated acute, active ongoing pollution) stops, lights and noise levels are reduced or removed entirely, the structure may not stand above water but see its vertical relief drastically cut down, and in some instances, the structure may even be relocated in areas where environmental conditions are drastically different. Given standing production platforms are inherently different from decommissioned structures, their environmental effects may not always be retained or directly transposable to decommissioned ones. Developing evidence to understand which effects (*benefits* or *harm*) are lost or retained following different decommissioning options can be key to support decision‐making regarding their end‐of‐life management. However, as of 2021, there was a notable paucity of empirical evidence describing how different decommissioning options might impact the environment (Lemasson et al., 2022, 2023). The clear mismatch between the empirical evidence from decommissioned structure that is needed and the available evidence identified in recent reviews (Lemasson et al., 2022, 2023) may limit our ability to reliably and accurately do so, hindering our ability to inform policy development and potential revision of legislation (Knights, Lemasson, Frost, & Somerfield, 2024; Paces et al., 2024).

To start overcoming this challenge, there is a clear need to carefully evaluate and synthesize the findings of the available empirical evidence base, however limited. To our knowledge, no synthesis of the environmental effects of decommissioned OG structures exists to date. Building on the scoping review of Lemasson et al. (2022), the present study systematically reviews the environmental effects of different decommissioning options for OG structures, using the available published peer‐reviewed evidence from global case studies of decommissioned structures. This state‐of‐the‐art assessment, accompanied by its fully coded evidence database, constitutes a valuable resource for researchers and decision‐makers to inform OG decommissioning.

## METHODS

This systematic review explores the environmental effects of OG decommissioning and expands on previous evidence synthesis work relating to offshore structures by Lemasson et al. (2021, 2022, 2024). The Collaboration for Environmental Evidence Guidelines and Standards for Evidence Synthesis in Environmental Management (2022) are applied as best as possible. For brevity, here we only briefly describe the methodology used to compile this systematic review, but full details are provided in Appendix S1 and a ROSES reporting standards form is provided in Lemasson and Knights (2026).

### Objective of the review and eligibility criteria

Our aim was to identify, describe, and synthesize the evidence of the ecological effects of the different decommissioning options used for offshore OG structures to answer: “what are the ecological effects on the marine environment of the different decommissioning options for oil and gas structures?” The following PICO components (Population‐Intervention‐Comparator‐Outcome, sensu CEE 2022) serve as the basis to our eligibility criteria:

1. *Population*: OG platform (sensu topside, jacket, foundation) in the marine environment (excluding cables, pipelines, and umbilicals, wells).
2. *Intervention*: Any decommissioning option (end‐of‐life management) used for OG structures.
3. *Comparator*(*s*): Any/all possible comparators (e.g., temporal comparators such as before/after; spatial comparators such as between different decommissioned structures or sites, between a decommissioned structure or site and a reference site, or a distance gradient away from a decommissioned structure or site; spatiotemporal comparators such as before‐after‐control‐impact [BACI] designs). We also considered studies without a strict comparator.
4. *Outcome*(*s*): All outcomes related to the ecology of the marine environment (e.g., diversity, population abundance, community structure, individual body size), except for outcomes relating to pollution.

Two additional components were considered: *Geographical scope*—global; *Type of study—*all empirical and observational field studies, that is, in situ case studies of decommissioned OG structures.

### Literature searches and database compilation

We used the systematic map database compiled by Lemasson et al. (2022) as our evidentiary starting point. That map (see Lemasson et al., 2021 for the protocol) identified and described the evidence base available at the time on the ecosystem effects of the presence and decommissioning of offshore structures in the sea. For this review, we filtered that database for studies on decommissioned OG structures.

In 2021, just 52 articles reported the effects of decommissioning OG structures (Lemasson et al., 2022). However, the imminent decommissioning challenge has introduced urgency in efforts to understand the ecological effects of different approaches. Consequently, it is likely that a crucial number of studies on the topic have been published since the literature searches by Lemasson et al. ended in 2021. Therefore, we performed an updated search in June 2024 using the same search strings as Lemasson et al. (2021, 2022) and retrieved articles from Web of Science Core Collection, Scopus, as well as the first 200 hits from Google Scholar.

### Eligibility criteria, article screening, and data extraction and coding

New articles retrieved from the updated searches were screened at title, abstract, and full‐text level to assess whether they met our eligibility criteria (see Appendix S1). Articles that did not contain relevant studies were rejected and the reason for rejection at full text recorded (see Lemasson & Knights, 2026). When a full text was not available, authors were contacted in the first instance. If no response was obtained, the article was rejected and the reason for rejection noted. Meta‐data (information describing a study) and effect data (here qualitative information relating to the effect of decommissioning on the marine environment) were extracted and coded for all articles retained in the final database (available online in Lemasson & Knights, 2026), following a standardized coding framework adapted from Lemasson et al. (2021). As the eligibility criteria for the systematic review are narrower than those used in Lemasson et al. (2021), full‐text screening was repeated for the subset of 52 articles selected from it to ensure their eligibility; meta‐data already coded were checked for correctness, and effect data were extracted and coded.

The final coded systematic review database after study appraisal and data extraction contains 42 unique articles relevant to our question and is included in the database in Lemasson and Knights (2026) (see also Appendix S1: Figure S1).

### Data synthesis and presentation

A given article may describe more than one study (a “study” referring to the unique combination of an intervention, population, and outcome).

The articles included in this systematic review are first described in a narrative synthesis of their characteristics (e.g., geographical distribution, type of decommissioning option considered, taxa, type of effect). Then, the effects reported (nature and direction) are synthesized narratively according to a strategy based mainly on the type of decommissioning option (Intervention category). Only findings from studies including a formal comparator are systematically reported in the narrative synthesis; findings from studies without comparators are not presented there unless directly pertinent and instead are mentioned in the *Discussion* section. A final subsection of the narrative synthesis of effects deals specifically with findings relating to non‐native species (NNS).

## REVIEW FINDINGS

### Nature and distribution of evidence (study characteristics)

#### Publication trend

In total, 146 studies from 42 articles were found on decommissioning. The earliest identified article was published in 1989 (Appendix S1: Figure S2) and just three further were published (two in 1994; one in 1997) until 2002, when a special issue on the topic of RtR was published in *ICES Journal of Marine Science* alongside one other publication elsewhere that resulted in five additional articles that year. However, mean numbers of yearly publications remained generally low until 2015 when numbers began to increase steadily year‐on‐year.

#### Geographical distribution

Articles contained studies on structures located across seven ocean basins and in the territorial waters of seven countries (Appendix S1: Table S2). The majority (59.5% of articles; *n* = 25) are in the GoM (USA) where OG activity is high and a RtR program is widely implemented. The North Sea ranked second with seven articles (16.7%; Norway [3], Netherlands [1], UK [3]). This low number is surprising given the number of platforms in the region that have already been decommissioned (OEUK, 2024). The Gulf of Thailand (GoT, Thailand) may represent an emerging hotspot for studies on OG decommissioning (all six articles [14.3%] were published from 2021 onward with studies beginning in 2018). It should be noted that, often, the studies emanating from one specific geographical area (GoM, Norway, GoT) were undertaken by similar authoring teams.

#### Decommissioning options (intervention type)

**FIGURE 1:**
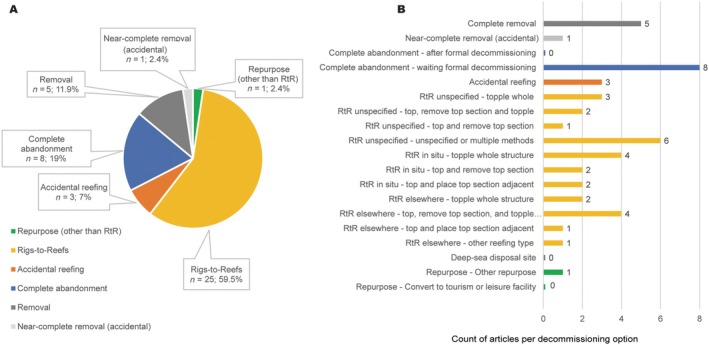
Distribution of the evidence by decommissioning options. (A) Grouped into six main categories. (B) All 17 specific interventions. Total percentage may exceed 100% as a single article can contain evidence on more than one decommissioning option. RtR: Rigs‐to‐Reefs. *n* = number of articles identified.

Effects of 17 different decommissioning options were identified, grouped into six main categories (Figure 1; Appendix S1: Table S3). Most articles report on RtR options (59.5%, *n* = 25), but with highly variable approaches (Figure 1B). Of note, authors did not always report clearly on the RtR methodology used, making it difficult to draw comparisons among studies. The next most frequent category relates to structures left standing whole in situ and awaiting formal decommissioning after cessation of operation (19% of articles, *n* = 8). Despite the number of structures globally that have already been fully removed at end‐of‐life, just 12% (*n* = 5) of articles report on the ecological effects of this decommissioning option. Other decommissioning options include accidental reefing due to hurricanes or accidental release during transport (7%, *n* = 3), and near‐complete removal after an accidental collision by a vessel (2.4%, *n* = 1). Despite being discussed and even known to occur in places (e.g., Seaventure Dive Rig, solar‐powered hydrogen production system; Alias & Go, 2023), repurposing a structure into something other than an artificial reef is not well reported, with only one study reporting on an obsolete structure being used as a search and rescue helicopter base (Fujii, 2015). We found no evidence on the effects of complete abandonment of a structure (standing whole in situ) following formal decommissioning (likely because it is currently not a legal option). Nor any studies on the effects of deep‐sea disposal, despite the option being commonly discussed in the early days of OG decommissioning.

#### Study designs and durations

**FIGURE 2:**
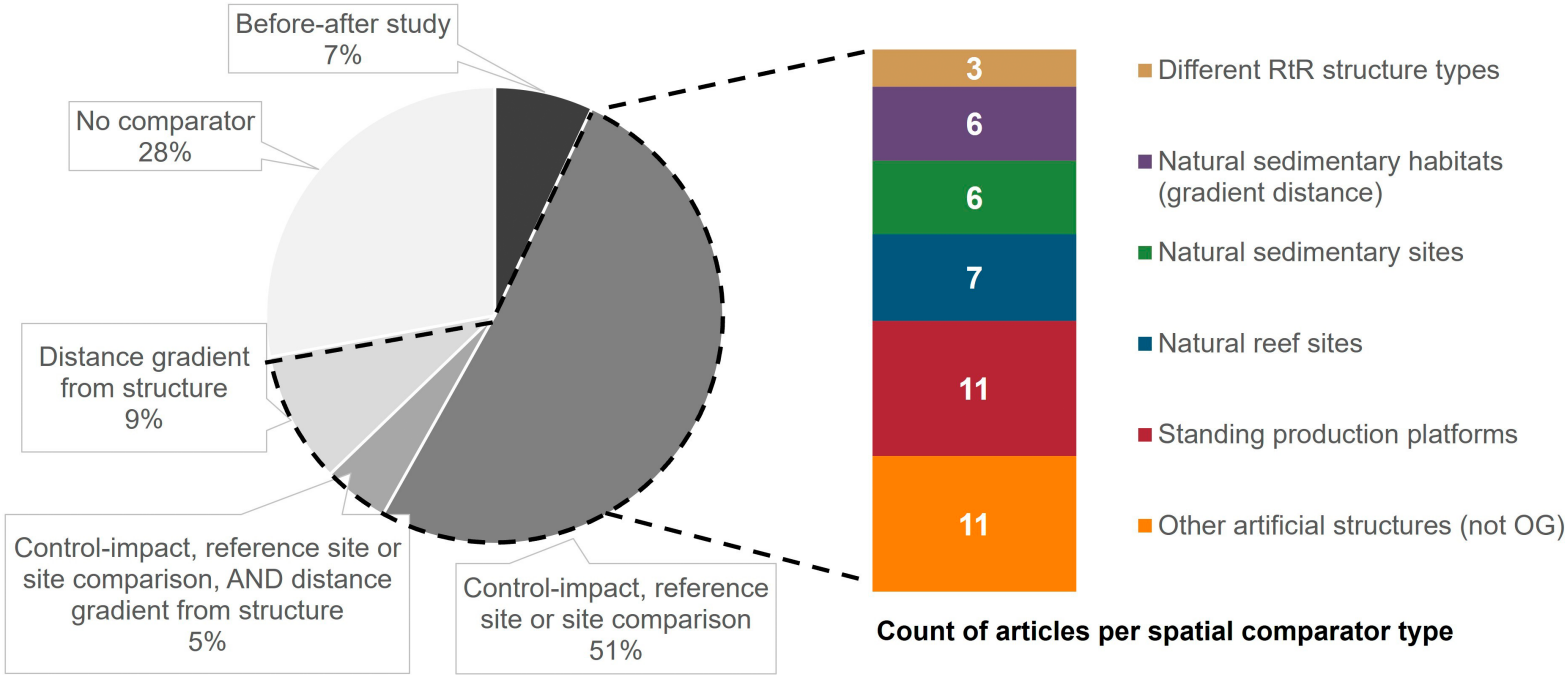
Distribution of the evidence by types of study designs and types of spatial comparators used. Total percentage may exceed 100% as a single article can contain multiple study designs. OG, oil and gas; RtR, Rigs‐to‐Reefs.

Only empirical or observational case studies of decommissioned structures were considered. Twelve articles (28%) reported on studies without comparator and simply described the decommissioned structures or sites. Formal comparators, either temporal or spatial, were used in 31 articles (74%) (Figure 2). Most considered spatial comparators (66% of articles) in the form of natural control/reference sites (e.g., natural reefs, natural sedimentary sites), other structures (site comparisons, other decommissioned structures, standing OG structures, other artificial structures), sampling along distance gradients away from the decommissioned site, or a combination of them (Figure 2; Appendix S1: Table S4). Just three articles (7%) contained studies using a temporal comparator (i.e., before‐after experimental design), and none using a BACI design.

The number of structures or sites surveyed (replication level) ranged from 1 (no replication) to 131. However, two studies that considered a very large number of structures (131 and 106, respectively) were observation‐only studies and did not provide structure‐specific data or undertake formal statistical comparisons. On this basis, they were removed from further assessment of study replication level. Average replication level was low (2.9 ± 2.5 SD). Most (58%, *n* = 14) were unreplicated or considered only two decommissioned sites (*n* = 8), eight studies (21%) assessed three decommissioned sites, and another eight studies (21%) considered four or more sites. Study duration spanned from 1 year (*n* = 20 articles) to 10 years (*n* = 1), but average duration was relatively short (2.2 years ±1.8 SD) (Appendix S1: Figure S3).

#### Taxonomic groups, and mention of commercial and non‐native species

**FIGURE 3:**
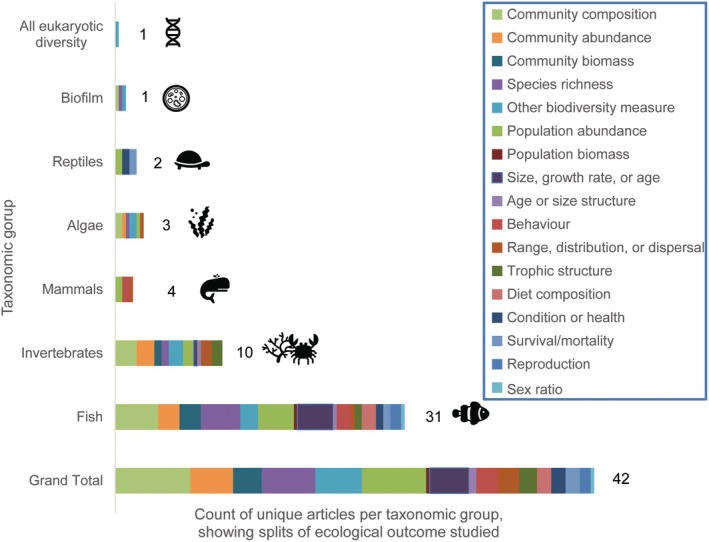
Count of unique articles per taxonomic groups, showing the split of articles by ecological outcome (effect) studied. A single article may contain evidence relating to more than one ecological outcome. Numbers next to the stacked columns represent the unique number of articles within each taxonomic group. The cumulative sum of articles across taxonomic groups may exceed the total number of unique articles included in the systematic review (*n* = 42) as a single article can contain evidence on more than one taxonomic group. Figure created by Anaëlle J. Lemasson using icons from Microsoft 365 Stock Images.

Most articles (74%, *n* = 31) report effects solely or partly on fish, followed by invertebrates (24%, *n* = 10) (Figure 3). There are few (or no) articles reporting the effects of decommissioning on other taxonomic groups (Figure 3). Of the 31 articles assessing fish, the majority surveyed all fish species present (*n* = 19). Most articles (52.4%, *n* = 22) reported on commercial species, mostly fish such as Red Snapper (*Lutjanus campechanus*) in the GoM (the focus, partly or solely, of 13 articles), and Cod (*Gadus morhua*), Haddock (*Melanogrammus aeglefinus*), and/or Saithe (*Pollachius virens*) in the North Sea (focus of four of the seven articles identified in this region). Only one article considered commercial invertebrates (Bomkamp et al., 2004, commercial crabs *Cancer antennarius*, *Cancer anthonyi*, and *Loxorhynchus grandis*). The occurrence of NNS was reported or investigated in eight articles (19%).

#### Outcomes (ecological effect types)

**FIGURE 4:**
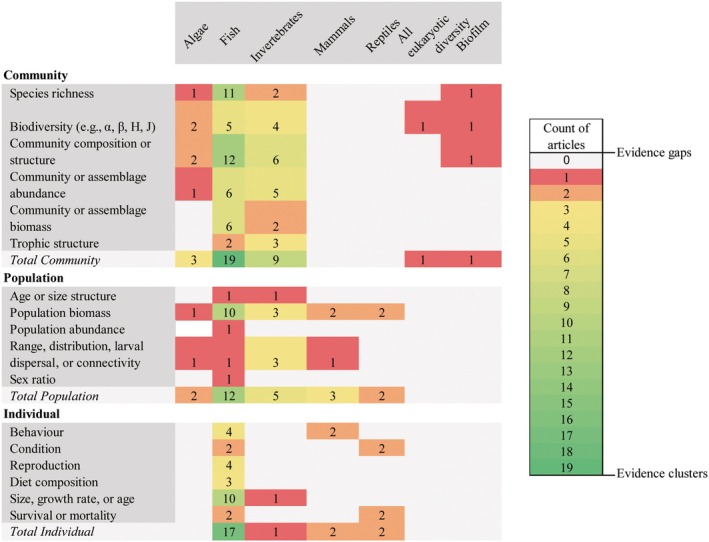
Heatmap illustrating the distribution of evidence across taxonomic groups and outcome categories. Evidence clusters are apparent for specific outcomes for fish (e.g., community composition, species richness, population biomass, individual size), and for invertebrates to a lesser extent (e.g., community composition, community biomass); evidence is scarce or nonexistent (i.e., evidence gaps) for other taxonomic groups across ecological outcomes.

The type of outcomes assessed are split into three main categories: effects at (1) community (27 articles), (2) population (19 articles), or (3) individual (20 articles) level (Figures 3 and 4; Appendix S1: Table S5). Studies often consider multiple effects across the three categories. Within each category, there are clear biases in terms of the type of effects assessed and the metrics measured. A focus on the effects on fish is apparent across community (19 of 27 articles), population (12 of 19 articles), and individual (17 of 20 articles) levels (Figure 4).

The most assessed community level outcomes are the effects on community composition/structure (40.5%; 17 articles), species richness (33.3%; 14 articles), other biodiversity metrics (23.8%; 10 articles), and abundance (26.2%; 11 articles) (Appendix S1: Table S5). Effects on trophic structure are comparatively less studied (12%; five articles) and largely relatively recent (published post‐2018). Population abundance, density, or percent cover were the most reported population‐level outcomes (36% of all articles) (Appendix S1: Table S5). Other population‐level metrics (age/size structure [4.8%], biomass [2%], range [9.5%], and sex ratio [2%]) were comparatively understudied. The most reported individual metric was size/growth rate/age (37%; 11 articles) of fish (10 of 11 articles relating to size/age). Five other metrics were also reported: individual behavior (12%), condition/health (9.5%), diet (9.5%), reproduction (7%), and survival/mortality (4.8%) (Figures 3 and 4; Appendix S1: Table S5).

### Ecological effects of different decommissioning options

#### Complete removal

Evidence describing the effects of the complete removal of structures on marine species, populations, and communities remains greatly limited. Globally, just five articles document effects, and of those, two were not designed to allow comparative assessment of effects (Gitschlag et al., 1997; Gitschlag & Herczeg, 1994). Moreover, each article focuses on a different taxonomic group (meiofauna, mobile macroinvertebrates, and marine mammals) and outcome variables, limiting our capacity to draw any broad conclusions. Structure removal in the GoM led to improved meiofaunal composition and increased abundance, closer to that of sedimentary reference sites than sites where platforms remained (Montagna et al., 2002). In contrast, for mobile macroinvertebrates associated with shell mounds off the coast of California, removal led to changes in species diversity and abundance which differed by trophic levels (Bomkamp et al., 2004). While in the short term the shell mound communities were found to persist in an altered state, over time they are expected to cease functioning. For marine mammals, changes in behavior and abundance (here, of the Harbour porpoise *Phocoena phocoena* in the North Sea) were only apparent during removal operations, and were comparable before and shortly after the removal of the structures (Fernandez‐Betelu et al., 2024), although it remains to be ascertained if and how long this legacy effect persists.

#### Near‐complete removal in situ (accidental)

In the North Sea, the gas platform Halfweg had ceased production and was waiting on complete removal when a ship collision meant the topside could be removed, but not all of the legs and the entire concrete gravity base. Coolen et al. (2020) found that the leftover structure had a notable positive effect on local biodiversity and macrofauna community structure, supporting 12 times more biomass than found on the surrounding flat sandy sediments. A total of 65 species were observed on what is a limited spatial extent, compared to 150 species observed within 30 km in the surrounding seabed, with only 10 species in common.

#### Complete abandonment—Leave standing in situ before formal decommissioning

Eight articles report the ecological effects of complete abandonment in situ (i.e., leaving the structure standing after cessation of operations). All structures had ceased production and were waiting formal decommissioning. Abandoned OG structures in the GoT were found to create complex 3‐D habitat that support complex and diverse biotic communities (Alexander et al., 2023; Harvey et al., 2021), whose fish assemblages significantly differed from that of natural sedimentary habitats, dominated by coral reef and coral reef‐associated species, and characterized by greater fish diversity (including unique species), abundance, and biomass (Alexander et al., 2022; Harvey et al., 2021). There, fish assemblages also differed with depths, with assemblages below 50 m being significantly different from that of shallower sections (Harvey et al., 2021). However, increased fish abundance is not always evidenced near structures (platform Albukjell 2/4F in the North Sea, Soldal et al., 2002), but video surveys and behavioral observations reveal fish aggregations (Albukjell 2/4F, Jørgensen et al., 2002; Løkkeborg et al., 2002; Soldal et al., 2002) and evidence mating and spawning behavior (Bigeye trevally *Caranx sexfasciatus* in the GoT, Madgett et al., 2022). Structures may also be used by marine mammals for foraging (Harbour porpoise off Scotland, Fernandez‐Betelu et al., 2022; but see Fernandez‐Betelu et al., 2024 for post‐removal effects).

#### Repurpose (other than RtR)

Just one article reports the effects of repurposing OG structures (Fujii, 2015). No comparator was used. The fish species composition and relative abundances at a large OG structure in the North Sea repurposed as a search and rescue helicopter base (standing whole in situ) varied with depths, with more fish caught near the bottom. Only six species were caught (which the authors suggest may likely be due to the use of baited traps), the three most abundant being commercially important species (cod, haddock, saithe).

#### Rigs‐to‐reefs

Thirty‐four articles report the outcomes of RtR. However, the exact reefing approach was highly variable with 11 variants of RtR (Figure 2), but each can be placed into one of two groupings: RtR options involving variants of toppling versus RtR options involving variants of topping. These two groupings are based on vertical relief (low relief: approximately horizontal to the seabed vs. high relief: structures left “standing” but with reduced height). Additionally, three articles assessed the effects of accidentally reefed platforms and can be considered a form of toppling.

##### RtR involving toppling

Fourteen articles investigated structures that had been toppled, either whole or after topping (with top section removed or placed adjacent), in situ or after relocation (Appendix S1: Table S3). All but two (Marnane et al., 2022; Sibley et al., 2023—GoT) were located in the GoM. Of note, several studies in the GoM focused wholly or partially on Red Snappers due to their economic importance there (Ajemian et al., 2015; Leontiou et al., 2021a, 2021b; Plumlee et al., 2020, 2021; Simonsen et al., 2015), and while outside the scope of this review, effects of decommissioning on this species may warrant further specific synthesis.

The habitat created by toppled platforms appears distinct from that created by other structures or by natural habitat. In the GoM, fish community composition and structure at toppled platforms was significantly different from standing production (Ajemian et al., 2015; Reynolds et al., 2018) and topped (Ajemian et al., 2015) platforms, from other types of ARs (Plumlee et al., 2020), and from natural sedimentary habitat away from the toppled structure (Reynolds et al., 2018). Toppled platforms support increased fish abundance and biomass compared to natural reef sites (Bollinger & Kline, 2017), as well as compared to further away from them (Reynolds et al., 2018), but similar abundance to topped and standing platforms (Ajemian et al., 2015). But effects on abundance compared to other ARs varied greatly across studies, likely depending on what the AR was made of (increased at toppled [Bollinger & Kline, 2017; Plumlee et al., 2020], similar [Ajemian et al., 2015], or reduced [Plumlee et al., 2020]). Effects on size in the GoM was less clear, and while fish size did not seem to differ between toppled structures and either standing platforms (Ajemian et al., 2015; Simonsen et al., 2015), topped platforms (Ajemian et al., 2015), other ARs (Ajemian et al., 2015), or natural reefs (Simonsen et al., 2015), these effects seemed to vary with age and maturity of the fish (Leontiou et al., 2021a, 2021b). In contrast, in the GoT, fish biomass and length at toppled platforms is comparable to a natural coral reef (Sibley et al., 2023).

Effects on fish diversity are less consistent, in some cases higher than on artificial or ship reefs (Plumlee et al., 2020), elsewhere similar to standing platforms and other types of RtR structures in some instances (Ajemian et al., 2015), or reduced compared to standing platforms (Reynolds et al., 2018). Species richness on a newly toppled jacket can be similar before (pre‐lift and wet tow) and after relocation and reefing while also rapidly colonized by new species not observed in its original site (Marnane et al., 2022). Marnane et al. recorded seven new species within 2 days of reefing, 16 “follower” species (predominantly reef‐associated) having followed the jacket from its operational area to the reefing site, but 10 pelagic species were lost.

Fish were also found to have a similar prey base at toppled platforms, standing production platforms, and natural reefs (although less diverse at toppled platforms), with no evidence of prey items specific to toppled structures (Simonsen et al., 2015). However, their diet appeared different at toppled platforms than at ARs of lower relief (Plumlee et al., 2021).

When comparing similar depth zones, epibenthic invertebrate communities growing on toppled structures were similar to those on topped platforms and standing production platforms (Rezek et al., 2018), but differed from surface communities (5 m) on standing platforms. Sammarco et al. (2014) also reported depth effects for coral communities, although the use of explosives during toppling contributed to the removal of the less resilient species. Nevertheless, epibenthos and coral total biomass and density appear similar between structure types (Rezek et al., 2018; Sammarco et al., 2014). Rezek et al. (2018) also reported that food web structure and trophic diversity were comparable across structures, suggesting that toppled platforms, as well as topped platforms, can retain the ecological function of standing platforms after reefing, but with a loss of shallow epibenthic communities. While no studies compared invertebrate diversity between structures, observations in the Mediterranean Sea of an accidentally reefed platform after 29 years (the *Paguro*, Ponti et al., 2002) indicate that toppled structures support a rich invertebrate assemblage of hard‐bottom species (53 species) that also varies in composition with depth and structural complexity.

Informal observations by Bull and Kendall (1994) support the idea that the toppling method influences fish and invertebrate communities, and also indicate that age is likely an important shaping factor. They observed that an older platform toppled “naturally” by a hurricane hosted more mature fish and invertebrate fouling communities, with higher abundances and species richness, over younger structures toppled using explosives.

##### RtR involving topping

Six articles reported on topped structures (bottom section left standing in situ or after relocation, either with its top section removed or placed adjacent) (Appendix S1: Table S3). All but one were in the GoM, where Seaman and Lindberg (1989) assessed structures that had been relocated from the GoM and reefed standing off the coast of Florida in the Atlantic. Studies assessed different outcomes on different populations, making it difficult to draw direct comparisons or firm conclusions. Evidence from studies comparing topped structures to toppled structures is already presented above in the *Rigs‐to‐reefs* section “RtR involving toppling” and will not be repeated here.

Early informal observations report topped platforms as hosts to a diverse assemblage of fish (47 species, across 20 families), suggested to be comparable to nearby natural reefs and ARs (Seaman & Lindberg, 1989). More recently, a study including a variety of formal comparators reported that topped structures had significantly different fish communities from toppled or deck platforms and from other types of artificial structures, despite having similar fish diversities. Fish communities (and their diversities) were similar to standing production platforms (Ajemian et al., 2015). In contrast, Johnston et al. (2022) reported a significant shift in fish community after topping, with differences in demersal and pelagic fish communities by depth, characterized by the loss of demersal fish originally associated with the now removed top section. The differences in fish community after topping likely caused the reported changes in fish trophic structure. Topped structures appear to retain a significant horizontal area of influence of approximately 20 m, where fish abundance and biomass are significantly increased compared to adjacent sedimentary habitats (Boswell et al., 2010). A vertical area of influence was also observed with depth, with greater densities, biomass, and sizes of fish at the bottom and midwater depths (Boswell et al., 2010).

One study investigated biofilms and found that distance to topped structures influences the diversity and community structure of biofilm developing on steel surfaces, but with clear differences between topped RtR and shipwrecks likely due to varying structure depths (Mugge et al., 2023).

##### RtR method unspecified, or multiple undifferentiated methods

Seven other articles reported the effects of RtR interventions, but with unclear or unspecified methods or included data pooled from structures decommissioned using different methods.

Krolow et al. (2022) found no difference in bony fish species richness or community composition between a reefed OG structure and a control sedimentary site; however, it differed and had lower species richness compared to ARs. Fish diversity on RtR can be similar to natural bank (reef) sites, with sites sharing common species (40% of species; Streich, Ajemian, Wetz, & Stunz, 2017). Yet, authors reported clear grouping of reef fish communities by habitat type, with communities at five R2R sites being similar to each other but significantly distinct from that of natural banks. Species abundances, however, may be highly variable both between and within habitats. In some cases, like the Red Snapper, abundances can be on average up to 7.8 times higher than on natural banks (Streich, Ajemian, Wetz, & Stunz, 2017), or can be no different (Streich, Ajemian, Wetz, Williams, et al., 2017). Fish mean length and mean age do not appear to differ between habitat/structure types, and all appear suitable for Red Snapper, but natural banks support greater proportions of older and larger individuals than standing and RtR platforms. Reefing may be effective at creating suitable habitat for Red Snapper feeding (Brewton et al., 2020; but with differences between RtR and natural reefs) and reproduction (Froehlich et al., 2021; no differences between RtR and ARs), although Schwartzkopf et al. (2017) suggests that RtR sites may be of lower nutritional “value” than natural sites.

#### Non‐native species

Eight articles reported effects or observations of the presence of NNS (Alexander et al., 2022, 2023; Coolen et al., 2020; Johnston et al., 2022; Sammarco et al., 2010, 2014; Streich, Ajemian, Wetz, & Stunz, 2017; Wanless et al., 2010).

Less than 8 months after its accidental stranding off the coast of Tristan da Cunha, the *A Turtle* OG rig was found to support an intact subtropical reef community typical of its location of origin, but largely comprising species of fish, invertebrates, and algae not native to Tristan da Cunha (62 NNS recorded across 14 Phyla and 40 families; Wanless et al., 2010).

In the GoM, survey records of 83 standing and toppled platforms reported the first record of the non‐native coral *Tubastraea micranthus* in the region (Sammarco et al., 2010). Although only observed on a single standing production platform, and not on any RtR toppled structures, its detection was deemed a potential threat to the GoM at the time. While surveying communities at platforms in the GoM, Sammarco et al. (2014) noted that the abundance of *Tubastraea coccinea*, a non‐native coral species closely related to *T. micranthus* and widely occurring in that area, was higher on toppled RtR platforms than on standing production platforms. This high frequency of occurrence was attributed to the use of explosives for toppling, as the species is known to flourish in disturbed habitats. Johnston et al. (2022) showed that *Tubastraea* sp. can even dominate the stony coral cover of platforms, both before and after topping (ca. 73% of cover), while native coral species were comparatively rare (27% of cover), further supporting the idea that its presence can lead to detrimental effects and should be closely monitored. Other NNS observed on RtR which may require monitoring include the Red Lionfish (*Pterois volitans*) (Streich, Ajemian, Wetz, & Stunz, 2017), although it was also observed on a natural bank, suggesting no habitat preference.

Two studies in the GoT used eDNA techniques to associate NNS with an area comprising both a decommissioned standing platform and a natural sedimentary habitat. In one study, no NNS were detected (Alexander et al., 2023). In another by the same author, three NNS of fish were detected in low abundance (the sea trout *Salmo trutta*, Nile tilapia *Oreochromis niloticus*, and the silverside *Argentina australiae*) (Alexander et al., 2022) but their location not specified making it impossible to make direct links to RtR.

In the North Sea, Coolen et al. (2020) reported occurrence in “low numbers” of two NNS (the amphipod *Monocorophium sextonae* and the colonial tunicate *Diplosoma listerianum*) on the remains of Halweg GBS (see report of other effects in *Near‐complete removal in situ [accidental*]).

## DISCUSSION

Whether artificial structures “benefit” the environment is still an ongoing debate, as the framing of expectations and outcomes is critical to our perspective of impacts. Prior to 2021, scientific understanding of the ecology of standing production platforms was available but restricted in focus to a few taxa and geographical extent (Fortune & Paterson, 2020; Lemasson et al., 2022), and evidence describing the aftereffects of decommissioning was limited at best (Fortune et al., 2024; Lemasson et al., 2022) and not yet synthesized, undermining the ability of policymakers and managers to make evidence‐informed decisions about which of the different decommissioning options might be best for nature and society.

**FIGURE 5:**
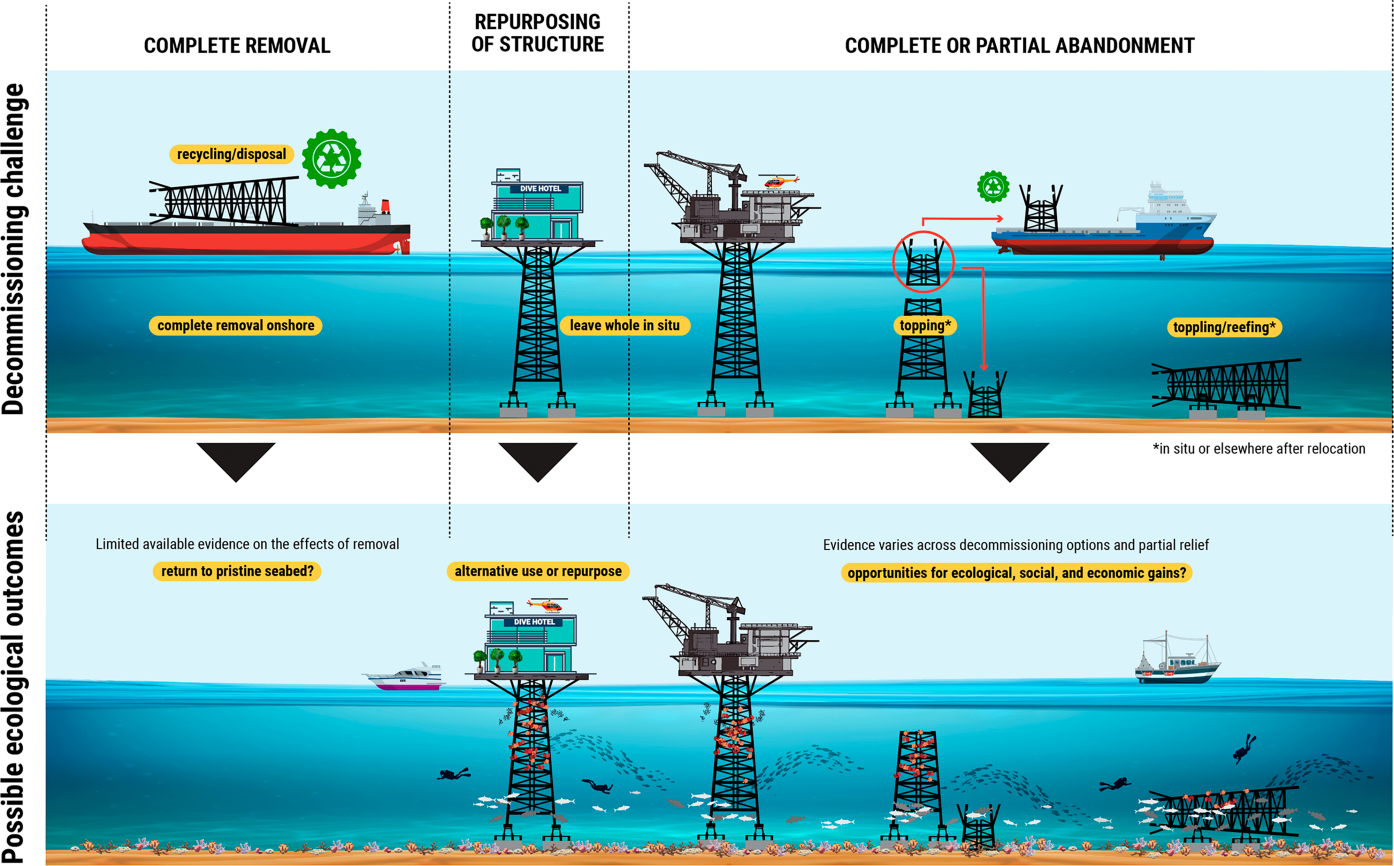
Visualization of the environmental effects of various decommissioning options for oil and gas platforms, based on this systematic evidence synthesis. Image created by Toco Creative Ltd. for the University of Plymouth.

Just 42 peer‐reviewed articles document the ecological effects of decommissioning OG platforms globally, highlighting a clear paucity of evidence to underpin decision‐making with respect to decommissioning. This is despite the enormous number of structures that have already been decommissioned (>5000 OG structures in the GoM alone as of 2017, Kaiser, 2022; Kaiser & Siddhartha, 2018), and the widely acknowledged decommissioning challenge societies are facing globally due to the many more still at sea rapidly approaching end‐of‐life (Knights, Lemasson, Frost, & Somerfield, 2024; Lemasson et al., 2023). Despite an increase in research on the topic in the last decade and mention of decommissioning in the literature (Fortune et al., 2024), likely reflecting the surge in RtR studies, the research effort to collect field data from decommissioned structures remains notably low. For instance, this mismatch is apparent for Australia: Indeed, we did not identify any case studies originating from this region despite decommissioning becoming a major topic of discussion there (Gissi et al., 2022; Koppel et al., 2023; MacIntosh et al., 2022; Melbourne‐Thomas et al., 2021; Sih et al., 2022). Given the active decommissioning research currently taking place in Australia, perhaps this mismatch is simply due to the often significant time lag between data collection and publication of findings. The limited amount of evidence available, coupled with the numerous variants of decommissioning methods used and the limitations of the experimental designs, means that drawing reliable comparisons between methods is challenging at best, if not nearly impossible. Additionally, given the clear research focus on fish with relatively little evidence on invertebrates or other taxonomic groups, any conclusions must be interpreted with caution, keeping in mind the significant gaps in knowledge. Below, we discuss the findings more broadly and their implications for end‐of‐life management of obsolete OG structures (Figure 5).

### Structures in or out?

Just five articles have considered the effects of the complete removal of OG platforms, greatly limiting our ability to draw conclusions (Figure 5). Recovery of seabed communities following the cessation of anthropogenic disturbances, such as aggregate extraction or bottom fishing, has been shown to occur (Froján et al., 2011; Simonini et al., 2007) but recovery time varies widely, sometimes not reached even after several decades (Foden et al., 2009; Knights et al., 2015). The highly limited evidence hints at a possible recovery of the seabed toward pre‐impacted condition over time after structure removal (Bomkamp et al., 2004; Montagna et al., 2002). However, if the objective of complete removal is a shift back to a “pristine” seabed state, this may not be a realistic objective due to other considerations such as legacy contamination from chronic hydrocarbon pollution, heavy metals such as mercury, and NORMS in offshore infrastructure (Gissi et al., 2022; MacIntosh et al., 2022) and the impacts that different removal stages may have on those contaminants (for instance through disturbance of sediments), not to mention due to the unavoidable shifting baseline syndrome (Pauly, 1995; Soga & Gaston, 2018).

Although not within the scope of this review, removing structures might provide benefits beyond the ecological value of regaining a “pristine” seabed. In specific regions, anything other than complete removal remains a complex political and societal topic (Jørgensen, 2012; Knights, Lemasson, Frost, & Somerfield, 2024; Techera & Chandler, 2015). Complete removal can free up valuable space for other maritime activities and industries, in what is an incredibly squeezed seascape (e.g., North Sea; Goodsir et al., 2015). Complete removal also removes navigational hazards and risks to other sea users, especially in areas of high spatial conflict (Szostek et al., 2025). On the other hand, if left in the water standing after cessation of operations, these complex 3‐D structures may “benefit” the local environment, with effects varying across regions. Similarly to what has been evidenced from standing production platforms (reviewed in Fortune et al., 2024), they can support fish communities more biodiverse and abundant than natural sedimentary habitats (Alexander et al., 2022; Harvey et al., 2021; but see Lemasson et al., 2024), provide habitat for their aggregation and reproduction (Fujii, 2015; Jørgensen et al., 2002; Madgett et al., 2022; Soldal et al., 2002), and can, in some instances, represent important foraging habitat for mammals (Fernandez‐Betelu et al., 2022, but see Fernandez‐Betelu et al., 2024).

The ongoing biodiversity crisis has been accelerated by habitat loss and modification (Chase et al., 2020; Riva & Fahrig, 2023; Sandor et al., 2022), including reducing connectivity. If structures can be used to introduce habitat and “restore” the connectivity that underpins healthy and sustainable ecological networks by re‐establishing dispersal pathways (James et al. 2025), this could rebalance perceptions of the “costs” of leaving structures in place. Indeed, the potential of OG platforms to influence connectivity (Coolen et al., 2020, but see Galaiduk et al., 2024 and McLean et al., 2022), act as de facto marine protected areas or refuge areas for species of conservation importance (Bergmark & Jørgensen, 2014), might be considered a compelling argument to overlook the negative impacts of structures remaining in place.

### Vertical or horizontal?

As our review brings to light, there is a variety of potential ways to decommission an OG structure at sea, often referred to as “reefing.” Reefing can be opted for multiple reasons and with different objectives, including intrinsic ecological value (biodiversity for the sake of biodiversity), creating social and recreational value (fishing and diving opportunities), or generating economic value (promoting commercially important species). Among many important factors influencing the ecological effects of reefed OG structures (e.g., age, structural complexity, location), vertical relief appears crucial (Figure 5).

Due to the conspicuous contrast in their vertical relief and depth, the habitats created by low‐relief reefed structures (with a horizontal orientation: toppled platforms) and high‐relief reefed structures (with a vertical orientation: standing but ceased operation, repurposed, or topped but standing) appear distinct (Ajemian et al., 2015; Reynolds et al., 2018). Standing at greater heights in the water column, high‐relief structures offer a greater variety of ecological niches (from increased structural complexity exposed to a wider range of environmental conditions) than low‐relief structures.

Both high‐relief (topped) and low‐relief (toppled) reefed structures host complex communities that are not only more diverse or abundant than natural sedimentary habitats (Reynolds et al., 2018; see also Lemasson et al., 2024) but also in some instances, equally or more diverse than natural reefs (Bollinger & Kline, 2017; Seaman & Lindberg, 1989; Sibley et al., 2023). The influence of the structures spatially extends both vertically through the water column (but richness has been shown to saturate 20 m off the bottom; Ajemian et al., 2015) and horizontally at distances away from them (20–40 m away; Boswell et al., 2010; Reynolds et al., 2018), a phenomenon known as the “halo effect.” Two individual studies have shown they can be more effective at providing ecological benefits than other types of artificial structures (Bollinger & Kline, 2017; Plumlee et al., 2020), although this contrasts with the global meta‐analysis findings of Lemasson et al. (2024), suggesting this might be a localized effect. Overall, both topped and toppled platforms may retain the ecological function of standing platforms after reefing, although topped platforms are generally more similar to standing platforms (Rezek et al., 2018), but with a loss of shallow (topped) or both shallow and midwater (toppled) communities associated with the change in vertical relief (Johnston et al., 2022; Rezek et al., 2018; Sammarco et al., 2014).

### Here or there?

The siting location of structures at sea may be a critical factor influencing their ability to generate beneficial ecological (as well as other) outcomes (Paxton et al., 2022). Local environmental conditions, such as depth, distance to the nearest coast or natural reef, and light regime, will dictate which species can colonize and which communities can be sustained locally. As such, the ecology and functioning of relocated reefed structures can be expected to differ from that of their original production site; which (*here or there*) is preferable and may lead to more beneficial ecological outcomes is yet to be understood. The current available literature, however, reveals a lack of evidence to inform this *here or there* question, as no studies formally compared the implications of relocating decommissioned OG structures to a new reefing site, to reefing them in situ. Perhaps of more importance to the decommissioning decision‐making process is the fact that reefing could be undertaken strategically to promote benefits locally (Knights, Lemasson, Frost, & Somerfield, 2024). Indeed, the location of the reefing site can in theory be selected to promote the development of particular species and communities, optimize specific ecological benefits, and achieve a priori objectives that may not be attainable (or desirable) at the original production site.

Relocation, however, may lead to unintended negative consequences. The relocation process itself may lead to the loss of epibenthic and mobile communities of potential ecological value that were associated with the structure at its production site, particularly if the relocation method involves a degree of emersion (Marnane et al., 2022). Perhaps of more importance, although outside the direct scope of this study, there is an emission cost to decommissioning, often overlooked but considered a priority research topic (Watson et al., 2023). The lifting and transport of structures, whether to a new reefing site or as part of complete onshore removal, can be an emission‐intensive process, releasing various toxic gases, greenhouse gases, and pollutants (Cantle & Bernstein, 2015; Smith & Byrd, 2021). Smith and Byrd (2021) estimated that decommissioning involving lifting and transport (in their study, transport to shore) may release 10‐times more emissions compared to in situ decommissioning (in their study, topping), an estimation roughly in agreement with Cantle and Bernstein (2015; 6.75× more pollution). At a time when societies' efforts are spent on reducing emissions, the carbon/emission footprint associated with the various decommissioning options should be considered in the decision‐making process (Davies & Hastings, 2023a, 2023b).

A major unintended consequence of relocation is associated with the introduction of NNS. Given the well‐documented ecological and economic costs of invasive species introductions in aquatic systems (Cuthbert et al., 2021), the significant concerns over the role OG structures play in allowing and promoting their spread are warranted (Braga et al., 2021). As evidenced in this study, decommissioned structures can be host to a variety of NNS (Coolen et al., 2020; Sammarco et al., 2010, 2014), which may or may not act as stepping stones through increased connectivity (McLean et al., 2022). The case study of the accidentally reefed *A Turtle* exemplifies the risks relocation can pose by inadvertently introducing these unwanted species to new locations (Wanless et al., 2010). If relocation is opted for, precaution ought to be adopted to mitigate the ecological and economic costs of NNS, including minimizing the risk of introduction through assessments and cleaning operations prior to reefing (Wanless et al. [2010] estimated that this could have prevented invasions and saved $20 million in management operations), as well as monitoring and early‐detection procedure post‐reefing which can be assisted by the use of novel methods such as eDNA metabarcoding (Alexander et al., 2022, 2023). Decision‐making about whether structures (if remaining in the sea) are relocated might depend on the wider costs, benefits, and risks of doing so on a case‐by‐case basis, as has been previously advocated (Knights, Lemasson, Firth, Beaumont, et al., 2024; Knights, Lemasson, Firth, Bond, et al., 2024; Knights, Lemasson, Frost, & Somerfield, 2024; Schroeder & Love, 2004).

### Which way forward?

Overall, the available evidence from decommissioned OG platforms, although limited, demonstrates that, if kept at sea, these structures can constitute “novel ecosystems” (sensu Hobbs et al., 2006; Van Elden & Meeuwig, 2019) that generate ecological value, such as habitat creation and local diversity increase. It is also apparent that standing inactive platforms and topped platforms may provide a greater level of benefits than toppled platforms, not only due to greater vertical relief, but also to an extent due to the detrimental effect of explosives still largely used for removal and toppling (Gitschlag et al., 1997; Gitschlag & Herczeg, 1994; Sammarco et al., 2014; see also Viada et al., 2008). However, our ability to draw firm conclusions and make recommendations is greatly limited by several aspects discussed below.

First, the numerous variations in decommissioning methods used (17 identified), particularly when dealing with reefing, make direct comparisons difficult. This is further hindered by often poorly reported clear methodological details with regard to the decommissioning procedure. Standardization in reporting, as advocated in other research fields (Haddaway & Macura, 2018), would go some way in overcoming this limitation.

Second, the diversity of experimental designs and comparators considered, along with low replication levels and short study duration, further complicates the picture. Although more recent studies do largely include formal comparators, these are in the majority of a spatial nature, comparing decommissioned OG to other sites or structures, with little temporal considerations providing baseline data prior to decommissioning (let alone prior to the siting of the OG structures in the first place). Designing studies with higher replication levels and longer duration, and including temporal comparators in the form of Before‐After or BACI, is thus recommended.

Third, we highlighted clear research biases (with efforts focused on fish and on a limited set of outcome measures), similar to biases identified in evidence syntheses on related topics (Fortune et al., 2024; Lemasson et al., 2022). There is an apparent need for studies to shift focus to depict a more comprehensive picture, for instance by incorporating measures beyond community composition and abundance or diversity‐based metrics such as functional traits (Madgett et al., 2023). Some recent papers identified in this review are paving the way by considering emerging metrics and using novel assessment techniques (eDNA, Alexander et al., 2022, 2023; Krolow et al., 2022; isotope analysis to assess diet and trophic function, Brewton et al., 2020; Schwartzkopf et al., 2017). Technological advancements will aid the process, allowing collection of a wider range of data and with higher accuracy (Bollinger & Kline, 2017; Bull et al., 2023; Reynolds et al., 2018; Sibley et al., 2023, 2024).

Additionally, the evidence and conclusions are bound by geographical context. Indeed, the majority of decommissioning research originates from the northern hemisphere, in particular from a single region (GoM), although the GoT appears as an emerging research spot. This leaves a clear gap in available information and understanding from elsewhere in the world that should be addressed by future research efforts. Local environmental context, in combination with local social, economic, and political factors such as recreational and economic activities, and local legislations, plays an important role in decommissioning considerations (Cripps & Aabel, 2002).

Finally, given the number of reported decommissioned OG structures (Gourvenec et al., 2022), one can question the reason for the paucity of evidence available from the peer‐reviewed published literature. Where are the post‐decommissioning monitoring data? Additional avenues may represent crucial sources of valuable evidence, including modeling and scenario‐based studies (Claisse et al., 2015; Meyer‐Gutbrod et al., 2020; Pondella et al., 2015; Tidbury et al., 2020), social and economic assessments (Cripps & Aabel, 2002; Kruse et al., 2015), multi‐attribute decision analyses (Henrion et al., 2015), as well as evidence from the gray literature (Szostek et al., 2024) or collected by industry—although access to these is commonly acknowledged to be challenging (Murray et al., 2018). Improved and coordinated international standardization of decommissioning procedures and follow‐up monitoring requirements, combined with mandatory open/accessible and transparent reporting of findings, will play a key role in overcoming many of the identified limitations and in generating usable data for assessment. This may be achieved through joint partnerships and establishment of common goals with industry, government agencies, or governing bodies giving green lights to decommissioning proposals, as well as other data holders—something increasingly advocated (Fortune et al., 2024; Lemasson et al., 2023; Murray et al., 2018).

Decommissioning is looming, and decisions must be taken, informed by best available evidence and driven by explicit objectives. It is increasingly recognized that this will be achieved on a case‐by‐case basis of benefits and costs trade‐offs which cannot be limited solely to the environment. When their decommissioning is designed and planned appropriately, leaving obsolete structures in the sea in one form or another may create opportunities for value co‐creation (Loia et al., 2021) across multiple sectors and for multiple actors through multifunctionality, such as for the fishing industry, recreational divers, and conservationists (Figure 5). However, while in some regions where structures are scarce (e.g., California, Australia) the contribution of each structure if left in the environment might be valuable, it may not be the case in regions where structures are numerous (e.g., GoM, North Sea). Environmental benefits from structures could be maximized further through ecological engineering and the application of nature‐inspired designs (Firth et al., 2024; Paxton et al., 2025; see discussion in Lemasson et al., 2024). This is not a new message. As early as 1989, Seaman and colleagues were proposing to modify rigs “before or after construction for biological purposes.” This idea has since received increasing consideration and even application for other offshore structures (reviewed in Pardo et al., 2023). However, caution must be applied to avoid greenwashing, unintended outcomes, and large‐scale abandonment (Firth et al., 2020, 2024). Importantly, while decommissioned OG structures could be reefed for conservation and restoration purposes, their reefing cannot replace active conservation and restoration measures of natural habitats (Paxton et al., 2025).

## AUTHOR CONTRIBUTIONS

Conceptualization: Anaëlle J. Lemasson, Antony M. Knights. Data curation: Anaëlle J. Lemasson. Formal analysis: Anaëlle J. Lemasson. Funding acquisition: Antony M. Knights, Anaëlle J. Lemasson. Methodology: Anaëlle J. Lemasson, Antony M. Knights. Visualization: Anaëlle J. Lemasson. Writing—original draft: Anaëlle J. Lemasson, Antony M. Knights. Writing—review and editing: Anaëlle J. Lemasson, Antony M. Knights.

## FUNDING INFORMATION

This work was supported by the UK Natural Environment Research Council and the INSITE Programme (INSITE SYNTHESIS project, grant number NE/W009889/1). Anaëlle J. Lemasson is also supported by a Research Culture Support Fellowship from the University of Plymouth.

## CONFLICT OF INTEREST STATEMENT

The authors declare no conflicts of interest.

## DATA AVAILABILITY STATEMENT

Data (Lemasson & Knights, 2026) are available in Figshare at https://doi.org/10.6084/m9.figshare.29390189.v1.

## Supporting information


Appendix S1.
