# Supplementary material for: What happens after oil and gas decommissioning? A global systematic review of marine environmental effects
Source: Ecol Appl. 2026 Apr 23;36:e70243. doi: 10.1002/eap.70243 (PMC13107095; doi:10.1002/eap.70243)
Supplement: Supplementary file 1 — Appendix S1. [file EAP-36-e70243-s001.pdf]

Appendix S1

What happens after oil and gas decommissioning? A global systematic review of marine environmental effects

Anaëlle J. Lemasson and Antony M. Knights

Ecological Applications

Contents

Section S1. Supplementary Methods **2**

S1.1. Objective of the Review *2*

S1.1.1. Primary objective and question 2

S1.1.2. Secondary question 2

S1.2. Brief summary of the methods used by Lemasson et al. (2021) *3*

S1.3. Compiling the systematic review database *3*

S1.3.1.Selection of studies from Lemasson et al. (2021) 3

S1.3.2.Update to the searches by Lemasson et al. (2021) 4

S1.3.3.Screening of articles from the updated searches 4

S1.3.4.Final screening of all articles added to the systematic review working database 4

S1.4. Study eligibility criteria *6*

S1.5. Critical appraisal and study validity assessment *7*

S1.6. Data coding and extraction strategy *7*

Section S2. Supplementary Results **9**

S2.1. Review descriptive statistics *9*

S2.2. Nature and distribution of evidence – supplementary figures and tables *9*

S2.2.1. Publication trend 9

S2.2.2. Geographical distribution 10

S2.2.3. Decommissioning options (Intervention type) 10

S2.2.4. Study designs and durations 12

S2.2.5. Outcome type (ecological effect) 14

Section S3. References **14**

Section S1. Supplementary Methods

S1.1. Objective of the Review

This systematic review is a continuation of the evidence synthesis work by Lemasson et al. (2021, 2022, 2024) on the environmental effects of offshore structures, including oil and gas. Their work, and therefore that of this review, follows the Collaboration for Environmental Evidence Guidelines and Standards for Evidence Synthesis in Environmental Management (2022) as best as possible.

S1.1.1 Primary objective and question

The primary objective of this systematic review is to identify, describe and synthesise the evidence of the ecological effects of the different decommissioning options used for offshore oil and gas structures. This primary objective is purposedly narrow in terms of its Population and Intervention components (*sensu* CEE 2022) as the review specifically is interested in specific offshore structures (oil and gas) and specific intervention type (options for decommissioning). However, the review is also broad in terms of Comparator and Outcome components (*sensu* CEE 2022), including studies that consider any types of comparators (as well as no comparator), and all types of ecological outcomes. The review is also of global geographical scope, given that decommissioning is a global challenge, and that case-studies reporting on decommissioning effects are known to be limited in numbers (A. Lemasson et al. 2022; A. J. Lemasson et al. 2023). The aim was thus to exhaustively harness and assess the available evidence on the topic.

Our primary question, linked to our primary objective, is therefore: What are the ecological effects on the marine environment of the different decommissioning options for oil and gas structures?”. This primary question has the following components:

Population Oil and gas platform (*sensu* topside, jacket, foundation) in the marine environment (excluding cables, pipelines and umbilicals, wells)

Intervention: Any decommissioning option (end-of-life management) used for oil and gas structures

Comparator(s): Any/all (e.g. before/after decommissioning; decommissioned structure or site/no structures or non-decommissioned structures). Also include studies that do not include comparators.

Outcome(s): all outcomes related to the ecology of the marine environment (e.g. diversity, population abundance, community structure, individual body size, etc.)

Type of study: all observational field studies i.e. in situ case-studies of “real-world” decommissioning of oil and gas structures.

S1.1.2. Secondary question

A secondary question of interest is linked with potential differences in effects between the different decommissioning options for oil and gas structures:

“Are there differences in effects on the marine environment between the various options for decommissioning oil and gas structures?”

However, due to the likely limited number of studies on the topic, we may not be able to answer partially or fully this secondary question.

This systematic review is a continuation of the evidence synthesis work by Lemasson et al. (2021, 2022, 2024) on the environmental effects of offshore structures, including oil and gas. Their work, and therefore that of this review, followed the Collaboration for Environmental Evidence Guidelines and Standards for Evidence Synthesis in Environmental Management (2022) as best as possible. We used the systematic map database compiled by Lemasson et al. (2022) as evidentiary basis and undertook a partial update in June 2024. Their systematic map, compiled using CEE guidelines (published protocol in Lemasson et al., 2021), identified and described the evidence base available at the time on the ecosystem effects of the presence and decommissioning of offshore structures in the sea. Therefore, for the purpose of this review we only require a subsample of that database consisting of studies on decommissioning oil and gas structures.

S1.2. Brief summary of the methods used by Lemasson et al. (2021)

The peer-reviewed published literature was searched systematically up to February 2021, and the full list of studies catalogued in the final map is freely available online. Details of the literature search and study selection steps behind the systematic map are available in the associated published protocol (Lemasson et al. 2021) and map report (Lemasson et al. 2022).

Briefly, a search string was entered into two bibliographic databases (Scopus, and Aquatic Sciences and Fisheries Abstracts), one platform (Web of Science Core Collection), one search engine (Google Scholar), and 10 institutional and organisational websites using the University of Plymouth subscription, in early 2021. Searches were undertaken in English only. ‘Snowballing’ of literature reviews was used to identify additional relevant literature. All references retrieved were managed using the reference manager Zotero. Articles were assessed for inclusion according to a 2-step hierarchical process: (1) at title and abstract level, and (2) at full text level, based on set agreed eligibility criteria. Meta-data (information describing each study) were extracted and coded for all articles retained in the final database, following a standardised coding framework.

S1.3. Compiling the systematic review database

S1.3.1. Selection of studies from Lemasson et al. (2021)

From the pool of articles collated in the systematic map, those relevant to this work were identified by selecting appropriate filters based on the selected PICO components detailed above and then collated in a new database. The process is illustrated in Figure S1. Specifically, we filtered and retained by: structure type, selecting only oil and gas; and by Intervention, retaining “decommissioning” and excluding those on their “alteration” or their “presence”. Through that process, 52 studies were identified. These studies, along with all metadata already

extracted by Lemasson et al., were then copied into a new database for the purpose of this systematic review.

S1.3.2. Update to the searches by Lemasson et al. (2021)

The field of research on offshore structures and their decommissioning is one that is rapidly accelerating, notably due to the imminent challenge that is their end-of-life management. Consequently, it is likely that a crucial number of studies on the topic has been published since the literature searches by Lemasson et al. ended in 2021. We thus undertook a partial update to these searches (see Figure S1).

To that end, we entered the same search strings used by Lemasson et al. into the following bibliographic databases: Web of Science Core Collection and Scopus, using the University of Plymouth subscription. We also aimed to retrieve the first 200 hits from Google Scholar. In deviation from what Lemasson et al. did, we did not search Aquatic Sciences and Fisheries Abstracts (due to access issues) nor did we search institutional and organisational websites (due to time constraints, but also to the low return of relevant hits that Lemasson et al. got from these sources). Searches were undertaken in English only. Searches were undertaken in June 2024.

S1.3.3. Screening of articles from the updated searches

For the purpose of this review, the retrieved articles were screened at title and abstract level (screen 1) as per criteria from Lemasson et al. (see Figure S1). At this stage, articles that passed this first screening were also tagged for the type of structures (oil and gas/artificial structure or reef/ other), and the intervention type (decommissioning/not decommissioning) they focussed on when the information was provided in the abstract (if not, they were tagged as "unspecified"). We did not screen at level 2 (full text with meta-data extraction) using the selection criteria by Lemasson et al., as our intention was not to formally update the systematic map at this stage. Instead, we rejected those articles that had passed the first stage of screening but clearly did not match our PICO components for this review. We instead selected those that passed and were tagged either "oil and gas", "artificial structure or reef" (this is because authors often refer to oil and gas structures as being artificial reefs), or "unspecified", as well as either "decommissioning" or "unspecified". These were then rapidly screened further at full-text (screen 2) to only gauge whether they met our Population and Intervention criteria for this review (i.e. a quick read for mention of oil and gas, and mention of decommissioning or end-of-life management); if they did, they were tagged accordingly and added to the systematic review working database for final full-text screening and meta-data and effect data extraction (mentioned above).

S1.3.4. Final screening of all articles added to the systematic review working database

A final screening (screen 3) was performed to ensure that all articles (and studies) added to the working database for meta-data and effect data extraction matched all PICO components and met the eligibility criteria (see Table S1 and Figure S1). As the eligibility criteria for the systematic review are narrower than those used in the systematic map, this additional screening was also

carried out on the articles selected from the systematic map at the study level. Articles were read at full text (if the full text was available) and the studies content checked against our eligibility criteria. Articles that did not contain relevant studies were rejected at this stage, and the reason for rejection recorded (see online supplementary database in Lemasson & Knights, 2026). All other articles were kept in the database for data extraction and coding (see below).

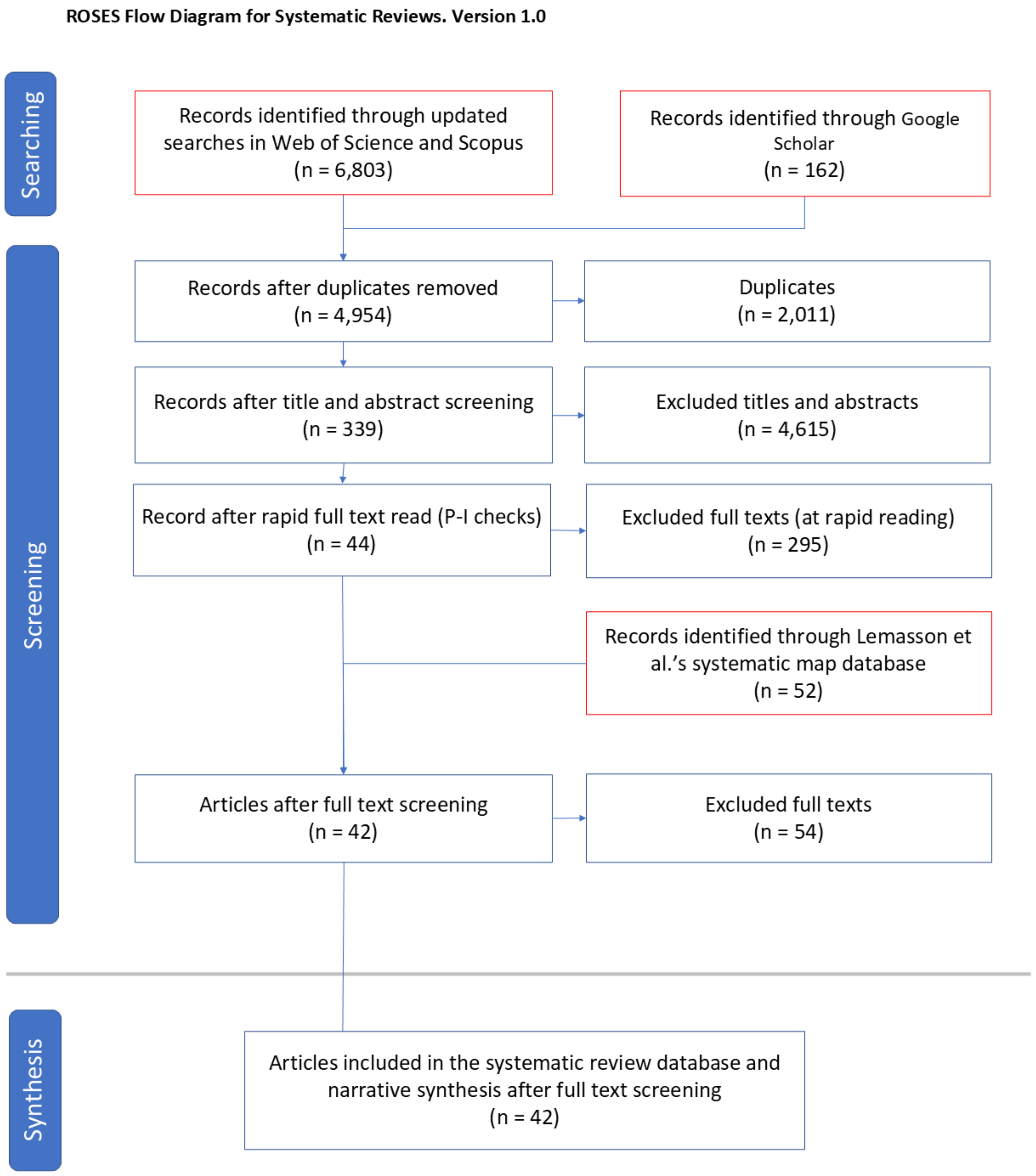

Figure S1: ROSES diagram detailing the systematic review process (literature search and article screening), with number of articles included and excluded at each stage. Red boxes highlight the various search origins of the articles.

S1.4. Study eligibility criteria

Final eligibility was assessed using the criteria listed in Table S1. These criteria are narrower than those used in the systematic map, and as such were applied to the entire working database of selected articles (from both new searches and the subset of the systematic map).

Table S1: List of criteria for inclusion

Include	Exclude
Population	
Any relevant components of the marine ecosystem. E.g. (but not limited to):<br>Ecosystems<br>Assemblages or communities<br>Populations or individual organisms<br>Habitats<br>Seabed or sediment (physical)<br>Water column<br><br>Geographical scope: global; but fully marine<br>Structure type considered: oil and gas platform (*sensu* topside, jacket, foundation)	Freshwater or estuarine environments<br>Other artificial structure types<br>Other infrastructure components associated with the OG industry (cables, pipelines, umbilicals, wells)
Intervention	
Any decommissioning option (end-of-life management) used for oil and gas structures. E.g. (but not limited to):<br>Complete removal<br>Leave as is in situ<br>Rigs-to-Reefs (R2R)<br>Repurpose (other than R2R)<br>Unintended decommissioning	Any other management intervention for oil and gas structures:<br>Construction/placing at sea (prior to production phase)<br>Presence/standing at sea (during production phase)<br>Alteration at sea (during production phase)
Comparator	
Any comparators<br>Temporal comparators (before/after, time series)<br>Spatial comparators (between different decommissioned structures or sites; between a decommissioned structure or site and a	

control, reference, or natural habitat site; distance gradient away from a decommissioned structure or site)<br>Spatiotemporal comparators (before-after-control-impact designs)<br>No strict comparator or correlative	
Outcome	
All possible ecological outcomes (any ecological effects on any components of the marine environment). E.g. (but not limited to):<br>Individual-level effects (such as on organism size, age, fertility, maturity, behaviour, condition…)<br>Population-level effects (such as on population abundance, population biomass, population age or size structure, sex ratio…)<br>Community/assemblage-level effects (such as species richness, diversity, community structure, trophic structure, community abundance, community biomass…)<br>Physical or chemical effects (such as sediment grain size, plastic pollution, flow…)	Outcome linked with:<br>pollution (such as hydrocarbon pollution, NORMS legacy, release of gases during decommissioning activities…)
Type of study	
Case study of decommissioned structures ("real-world" studies that have tested, measured, or assessed effects)	Anything else (laboratory-based, modelling, conceptual, framework, reviews, social or perception studies, cost-benefit analyses, etc.)
Other	
Journal articles in English	Anything else

S1.5. Critical appraisal and study validity assessment

The studies were critically appraised using the criteria described in Table S1 to evaluate their internal validity. Note that a single article can describe more than one study and allocated each article included given a unique identifier. Here we assessed the validity per article, and only findings from studies including a formal comparator are reported in the narrative synthesis, while other studies identified without a comparator will be discussed only when pertinent, or in the Discussion section.

S1.6. Data coding and extraction strategy

Meta-data (information describing each study) and effect data (here qualitative information relating to the effect(s) of decommissioning on the marine environment) were extracted and coded for all articles retained in the final database (Figure S1; online supplementary database in Lemasson & Knights, 2026), following a standardised coding framework. This framework was amended as needed from that used in the systematic map by Lemasson et al.

For the subset of articles obtained from the systematic map, the meta-data already coded were checked for correctness, and effect data extracted and coded. For the articles newly added (from the 2024 searches), all meta-data and effect data were extracted and coded into the database. During data extraction, the missing or unclear information was coded as such. While the meta-data from the subset of articles in the systematic map was extracted and coded by a team of reviewers, any additional data (meta-data and effect data) coded since was done by one reviewer only (AJL). Although a single article can describe more than one study, here we extracted the data per article.

The coded systematic review database is included in the online database in Lemasson & Knights (2026).

Section S2. Supplementary Results

S2.1. Review descriptive statistics

By applying the relevant filters to the systematic map database, 51 articles were identified as potentially containing relevant information on the environmental effects of decommissioning oil and gas structures. An additional article listed in the map but not under "decommissioning" (by error) was also identified and included. The search update returned 6,965 potential records (2,561 from Web of Science Core Collection; 4,242 from Scopus; 162 from Google Scholar). These new records were deduplicated, removing 2,011 records. Amongst the 4,954 unique articles remaining, 339 were kept following screen 1 at abstract and title level. Of these, 44 articles were retained as likely containing relevant effect data after screen 2 (rapid full text read to gauge Population and Intervention criteria). The working database after screen 2 thus contained 96 unique articles (52 from the systematic map, 44 from the updated searches). Of these, 54 were rejected during screen 3 because they did not meet our full set of criteria (did not match the PICO components). The final working database after study appraisal, data extraction and coding therefore contained 42 unique articles (see Figure S1 and online supplementary database in Lemasson & Knights, 2025). Note that while here we report on the number of unique articles retrieved, a given article may describe more than one study (a "study" referring to the unique combination of an intervention, population, and outcome).

S2.2. Nature and distribution of evidence – supplementary figures and tables

S2.2.1. Publication trend

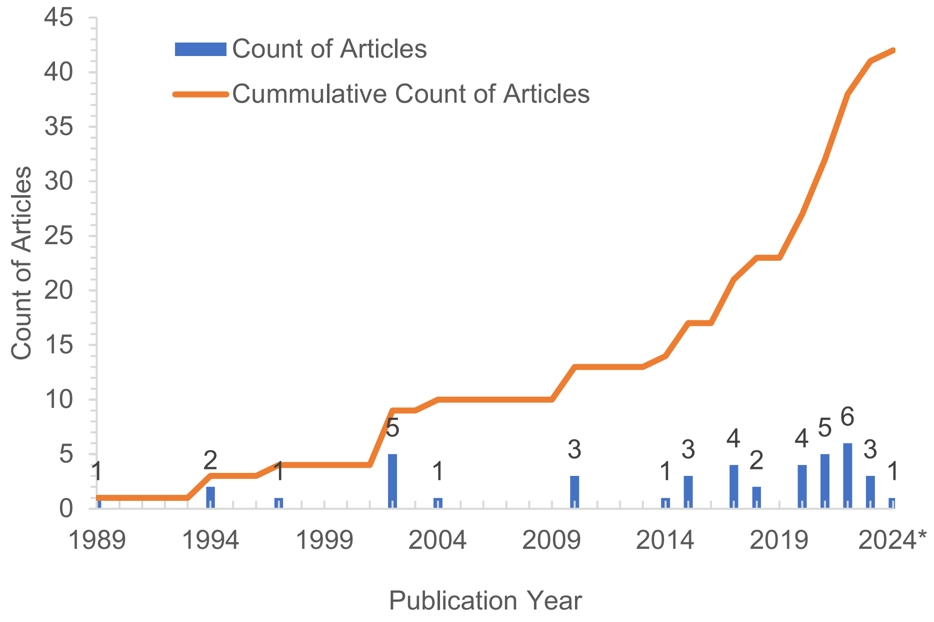

Figure S2: Publication trend of articles assessing the ecological effects of decommissioned oil and gas platforms. *Note that 2024 was an incomplete search year (until June).

S2.2.2. Geographical distribution

Table S2: Geographical spread of identified articles by ocean basin and by country, with associated references

Ocean Basin<br>Country	Count of Articles	References
**South-central Atlantic**	**1**	
British Overseas Territory	1	Wanless et al. 2010
**Gulf of Mexico**	**25**	
USA	25	Sammarco et al. 2010; Boswell et al. 2010; Bull & Kendall 1994; Bull et al. 2023; Montagna et al. 2002; Simonsen et al. 2015; Reynolds et al. 2018; Wets et al. 2020; Gitschlag et al. 1997; Sammarco et al. 2014; Bollinger et al. 2017; Streich et al. 2017a; Schwartzkopf et al. 2017; Brewton et al. 2020; Gitschlag & Herczeg 1994; Ajemian et al. 2015; Rezek et al. 2018; Streich et al. 2017b; Mugge et al. 2023; Johnston et al. 2022; Plumlee et al. 2021; Leontiou et al. 2021a; Leontiou et al. 2021b; Froehlich et al. 2021; Krolow et al. 2022; Plumlee et al. 2020
**Gulf of Thailand**	**6**	
Thailand	6	Madgett et al. 2022; Sibley et al. 2023; Harvey et al. 2021; Alexander et al. 2022; Marnane et al. 2022; Alexander et al. 2023
**Mediterranean Sea**	**1**	
Italy	1	Ponti et al. 2002
**North Sea**	**7**	
Norway	3	Soldal et al. 2002; Jørgensen et al. 2002; Løkkeborg et al. 2002
The Netherlands	1	Coolen et al. 2020
UK	3	Fujii 2015; Fernandez-Betelu et al. 2022; Fernandez-Betelu et al. 2024
**North-east Pacific**	**1**	
USA	1	Bomkamp et al. 2004
**North-west Atlantic**	**1**	
USA	1	Seaman et al. 1989

S2.2.3. Decommissioning options (Intervention type)

Table S3: Type of decommissioning options, with count of articles per category and sub-category, and associated references. Note that some options were identified from the literature, but not

studies specifically reporting ecological effects were found. Total count of articles may exceed 42 as some articles included information on more than one decommissioning option.

Decommissioning option	Count of articles	References
**Removal**	**5**	
Complete removal	5	Montagna et al. 2002; Bomkamp et al. 2004; Gitschlag & Herczeg 1994; Gitschlag et al. 1997; Fernandez-Betelu et al. 2024
**Complete abandonment**	**8**	
Leave standing in situ after cessation of operations, waiting formal decommissioning	8	Soldal et al. 2002; Løkkeborg et al. 2002; Jørgensen et al. 2002; Madgett et al. 2022; Fernandez-Betelu et al. 2022; Harvey et al. 2021; Alexander et al. 2022; Alexander et al. 2023
Leave standing in situ after formal decommissioning	0	
**Deep-Sea Disposal**	**0**	
Remove, transport, and dump at a deep-sea disposal site	0	
**Repurpose (other than R2R)**	**1**	
Convert to tourism or leisure facility	0	
Other repurpose	1	Fujii 2015
**Rigs-to-Reefs**	**25**	
R2R elsewhere - other reefing type	1	Ajemian et al. 2015
R2R elsewhere - top and place top section adjacent	1	Seaman et al. 1989
R2R elsewhere - top, remove top section, and topple bottom section	4	Bull & Kendall 1994; Plumlee et al. 2021; Leontiou et al. 2021; Plumlee et al. 2020
R2R elsewhere - topple whole structure	2	Sibley et al. 2023; Marnane et al. 2022
R2R in situ - top and place top section adjacent	2	Boswell et al. 2011; Mugge et al. 2023
R2R in situ - top and remove top section	2	Ajemian et al. 2015; Johnston et al. 2022
R2R in situ - topple whole structure	4	Sammarco et al. 2010; Bull & Kendall 1994; Reynolds et al. 2018; Simonsen et al. 2015
R2R unspecified if in situ or elsewhere - method unspecified or multiple undifferentiated methods	6	Streich et al. 2017a; Schwartzkof et al. 2017; Streich et al. 2017b; Krolow et al. 2022; Froehlich et al. 2021; Brewton et al. 2020; Wetz et al. 2020
R2R unspecified if in situ or elsewhere - top and remove top section	1	Rezek et al. 2018
R2R unspecified if in situ or elsewhere - top, remove top section and topple	2	Bollinger & Kline 2017; Leontiou et al. 2021
R2R unspecified if in situ or elsewhere - topple whole	3	Rezek et al. 2018; Ajemian et al. 2015; Sammarco et al. 2014

**Accidental reefing**	**3**	
Accidental reefing (e.g. during transport, following hurricane...)	3	Wanless et al. 2010; Bull & Kendall 1994; Ponti et al. 2002
**Accidental near-complete removal**	**1**	
Near-complete removal/partial abandonment in situ (accidental)	1	Coolen et al. 2020

S2.2.4. Study designs and durations

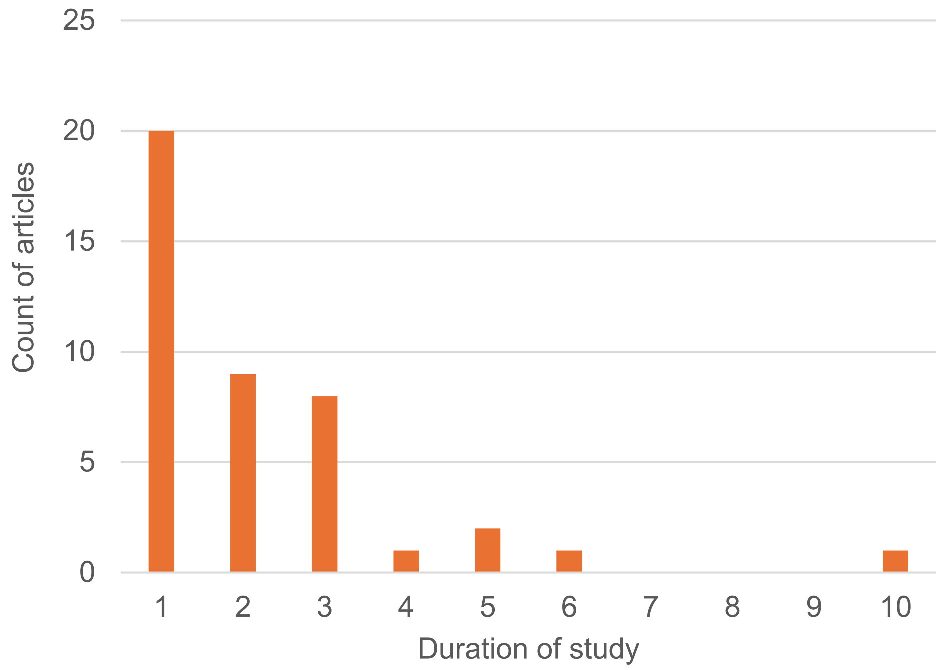

Figure S3: Count of articles identified in the literature by study duration in years. Average study duration was 2.2 years (±1.8 SD).

Table S4: Types of spatial comparators used in the identified studies, and count of articles with their associated reference.

Type of comparators considered	Count of articles	References
Different R2R structure types AND other artificial structures (not OG) AND natural reef sites	1	Bollinger & Kline, 2017
Different R2R structure types AND standing production platforms AND other artificial structures (not OG)	1	Ajemian et al. 2015
Different R2R structure types AND standing production platforms	1	Rezek et al. 2018
Natural reef sites	3	Streich et al. 2017a; Schwartzkopf et al. 2017; Sibley et al. 2023
Natural sedimentary habitats (gradient distance)	4	Boswell et al. 2010; Soldal et al. 2002 ; Løkkeborget al. 2002 ; Ferandez-Betelu et al. 2022
Natural sedimentary sites	4	Coolen et al. 2020; Harvey et al. 2021 ; Alexander et al. 2022; Alexander et al. 2023
Other artificial structures (not OG)	3	Froehlich et al. 2021; Plumlee et al. 2021 ; Plumlee et al. 2020
Other artificial structures (not OG) AND natural sedimentary habitats (gradient distance)	1	Mugge et al. 2023
Other artificial structures (not OG) AND natural sedimentary sites	1	Krolow et al. 2022
Standing production platforms AND other artificial structures (not OG)	2	Leontiou et al. 2021a,b
Standing production platforms AND other artificial structures (not OG) AND natural reef sites	1	Simonsen et al. 2015
Standing production platforms AND other artificial structures (not OG) AND natural sedimentary sites	1	Montagna et al. 2002
Standing production platforms	1	Sammarco et al. 2014
Standing production platforms AND natural reef sites	2	Brewton et al. 2020; Streich et al. 2017b
Standing production platforms AND natural sedimentary habitats (gradient distance)	1	Reynolds et al. 2018
Standing production platforms AND natural sedimentary sites	1	Bomkamp et al. 2004

S2.2.5. Outcome type (ecological effect)

Table S5: Type of ecological effects (or metrics) reported in the identified articles. Note that the total count of articles may exceed 42 as some articles included information on more than one outcome (effect) type.

Outcome level	Count of articles
**Community**	***27***
Community composition or structure	17
Species richness	14
Community/assemblage abundance (total fish, MaxN, epifauna…)	11
Biodiversity (alpha, beta, gamma, H, J, Δ)	10
Community/assemblage biomass	8
Trophic structure	5
**Population**	***19***
Population abundance, density, or % cover	15
Range, distribution, or larval dispersal/connectivity	4
Age or size structure	2
Population biomass	1
Sex ratio	1
**Individual**	***20***
Size, growth rate, or age	11
Behaviour (reproductive, avoidance, migration, use, foraging)	5
Diet composition	4
Condition, health, or injury	4
Fecundity, Maturity, Reproductive output, egg/sperm quality, or recruitment	3
Survival/mortality	2

Section S3. References

Ajemian, Matthew J., Jennifer J. Wetz, Brooke Shipley-Lozano, J. Dale Shively, and Gregory W. Stunz. 2015. 'An Analysis of Artificial Reef Fish Community Structure along the Northwestern Gulf of Mexico Shelf: Potential Impacts of "Rigs-to-Reefs" Programs'. *PloS One* 10 (5): e0126354.

Alexander, JB, MJ Marnane, TS Elsdon, M Bunce, P Sitaworawet, S Songploy, S Chaiyakul, and ES Harvey. 2023. 'Using Environmental DNA to Better Inform Decision Making around Decommissioning Alternatives for Offshore Oil and Gas Infrastructure'. *SCIENCE OF THE TOTAL ENVIRONMENT* 901 (November). https://doi.org/10.1016/j.scitotenv.2023.165991.

Alexander, JB, MJ Marnane, TS Elsdon, M Bunce, S Songploy, P Sitaworawet, and ES Harvey. 2022. 'Complementary Molecular and Visual Sampling of Fish on Oil and Gas Platforms Provides

Superior Biodiversity Characterisation'. *MARINE ENVIRONMENTAL RESEARCH* 179 (July). https://doi.org/10.1016/j.marenvres.2022.105692.

Bollinger, Michael A., and Richard J. Kline. 2017. 'Validating Sidescan Sonar as a Fish Survey Tool over Artificial Reefs'. *Journal of Coastal Research* 33 (6): 1397–1407.

Bomkamp, R. E., H. M. Page, and J. E. Dugan. 2004. 'Role of Food Subsidies and Habitat Structure in Influencing Benthic Communities of Shell Mounds at Sites of Existing and Former Offshore Oil Platforms'. *Marine Biology* 146 (1): 201–11. https://doi.org/10.1007/s00227-004-1413-8.

Boswell, Kevin M., R. J. Wells, James H. Cowan Jr, and Charles A. Wilson. 2010. 'Biomass, Density, and Size Distributions of Fishes Associated with a Large-Scale Artificial Reef Complex in the Gulf of Mexico'. *Bulletin of Marine Science* 86 (4): 879–89.

Brewton, Rachel A., Charles H. Downey, Matthew K. Streich, Jennifer J. Wetz, Matthew J. Ajemian, and Gregory W. Stunz. 2020. 'Trophic Ecology of Red Snapper Lutjanus Campechanus on Natural and Artificial Reefs: Interactions between Annual Variability, Habitat, and Ontogeny'. *Marine Ecology Progress Series* 635:105–22.

Bull, Ann, and James J. Kendall Jr. 1994. 'An Indication of the Process: Offshore Platforms as Artificial Reefs in the Gulf of Mexico'. *Bulletin of Marine Science* 55 (2–3): 1086–98.

Bull, AS, MM Nishimoto, MS Love, S Clark, K Seeto, M McCrea, C Park, et al. 2023. 'Comparison of Methods (ROV, Diver) Used to Estimate the Composition and Abundance of Biota Colonizing an Offshore Oil Platform: A Pilot Study'. *CONTINENTAL SHELF RESEARCH* 252 (January). https://doi.org/10.1016/j.csr.2022.104856.

Collaboration for Environmental Evidence. 2022. 'Guidelines and Standards for Evidence Synthesis in Environmental Management. Version 5.1'. AS Pullin, GK Frampton, B Livoreil & G Petrokofsky, Eds. www.environmentalevidence.org/information-for-authors.

Coolen, Joop WP, Oliver Bittner, Floor MF Driessen, Udo van Dongen, Midas S. Siahaya, Wim de Groot, Ninon Mavraki, Stefan G. Bolam, and Babeth van der Weide. 2020. 'Ecological Implications of Removing a Concrete Gas Platform in the North Sea'. *Journal of Sea Research* 166:101968.

Fernandez-Betelu, O., I.M. Graham, F. Malcher, E. Webster, S.-H. Cheong, L. Wang, V. Iorio-Merlo, S. Robinson, and P.M. Thompson. 2024. 'Characterising Underwater Noise and Changes in Harbour Porpoise Behaviour during the Decommissioning of an Oil and Gas Platform'. *Marine Pollution Bulletin* 200. https://doi.org/10.1016/j.marpolbul.2024.116083.

Fernandez-Betelu, Oihane, Isla M. Graham, and Paul M. Thompson. 2022. 'Reef Effect of Offshore Structures on the Occurrence and Foraging Activity of Harbour Porpoises'. *Frontiers in Marine Science* 9:980388.

Froehlich, CYM, AM Lee, R Oquita, CE Cintra-Buenrostro, and JD Shively. 2021. 'Reproductive Characteristics of Red Snapper Lutjanus Campechanus on Artificial Reefs in Different Jurisdictions'. *REGIONAL STUDIES IN MARINE SCIENCE* 47 (September). https://doi.org/10.1016/j.rsma.2021.101936.

Fujii, Toyonobu. 2015. 'Temporal Variation in Environmental Conditions and the Structure of Fish Assemblages around an Offshore Oil Platform in the North Sea'. *Marine Environmental Research* 108:69–82.

Gitschlag, G. R., and A. Herczeg. 1994. 'Explosive Removals of Energy Structures'. *Marine Fisheries Review* 56:1.

Gitschlag, Gregg R., Bryan A. Herczeg, and Theresa R. Barcak. 1997. 'Observations of Sea Turtles and Other Marine Life at the Explosive Removal of Offshore Oil and Gas Structures in the Gulf of Mexico'. *Gulf and Caribbean Research* 9 (4): 247–62.

Harvey, ES, SL Watts, BJ Saunders, D Driessen, LAF Fullwood, M Bunce, S Songploy, et al. 2021. 'Fish Assemblages Associated With Oil and Gas Platforms in the Gulf of Thailand'. *FRONTIERS IN MARINE SCIENCE* 8 (September). https://doi.org/10.3389/fmars.2021.664014.

Henrion, Max, Brock Bernstein, and Surya Swamy. 2015. 'A Multi-Attribute Decision Analysis for Decommissioning Offshore Oil and Gas Platforms'. *Integrated Environmental Assessment and Management* 11 (4): 594–609.

Hobbs, Richard J., Salvatore Arico, James Aronson, Jill S. Baron, Peter Bridgewater, Viki A. Cramer, Paul R. Epstein, et al. 2006. 'Novel Ecosystems: Theoretical and Management Aspects of the New Ecological World Order'. *Global Ecology and Biogeography* 15 (1): 1–7. https://doi.org/10.1111/j.1466-822X.2006.00212.x.

Johnston, MA, MF Nuttall, EL Hickerson, K O'Connell, RD Blakeway, JA Embesi, J MacMillan, D Peter, and GP Schmahl. 2022. 'Characterizing the Biological Community before and after Partial Removal of an Offshore Gas Platform in the Northwestern Gulf of Mexico'. *ENVIRONMENTAL MANAGEMENT* 70 (6): 1078–92. https://doi.org/10.1007/s00267-022-01714-8.

Jørgensen, Terje, Svein Løkkeborg, and Aud Vold Soldal. 2002. 'Residence of Fish in the Vicinity of a Decommissioned Oil Platform in the North Sea'. *ICES Journal of Marine Science* 59 (suppl): S288–93.

Krolow, AD, AD Geheber, and KR Piller. 2022. 'If You Build It, Will They Come? An Environmental DNA Assessment of Fish Assemblages on Artificial Reefs in the Northern Gulf of Mexico'. *TRANSACTIONS OF THE AMERICAN FISHERIES SOCIETY* 151 (3): 297–321. https://doi.org/10.1002/tafs.10352.

Lemasson, AJ, AM Knights, M Thompson, G Lessin, N Beaumont, C Pascoe, AM Queirós, L McNeill, M Schratzberger, and PJ Somerfield. 2021. 'Evidence for the Effects of Decommissioning Man-Made Structures on Marine Ecosystems Globally: A Systematic Map Protocol'. *ENVIRONMENTAL EVIDENCE* 10 (1). https://doi.org/10.1186/s13750-021-00218-y.

Lemasson, AJ.; Knights, AM. 2026. Supplementary files (database and ROSES form) to *What happens after oil and gas decommissioning? A global systematic review of marine environmental effects*. figshare. Dataset. https://doi.org/10.6084/m9.figshare.29390189

Lemasson, A.J., P.J. Somerfield, M. Schratzberger, and A.M. Knights. 2023. 'Challenges of Evidence-Informed Offshore Decommissioning: An Environmental Perspective'. *Trends in Ecology and Evolution* 38 (8): 688–92. https://doi.org/10.1016/j.tree.2023.04.003.

Lemasson, AJ, PJ Somerfield, M Schratzberger, CL McNeill, J Nunes, C Pascoe, SCL Watson, MSA Thompson, E Couce, and AM Knights. 2022. 'Evidence for the Effects of Decommissioning Man-Made Structures on Marine Ecosystems Globally: A Systematic Map (Vol 11, 35, 2022)'. *ENVIRONMENTAL EVIDENCE* 11 (1). https://doi.org/10.1186/s13750-022-00293-9.

Lemasson, A.J., P.J. Somerfield, M. Schratzberger, M.S.A. Thompson, L.B. Firth, E. Couce, C.L. McNeill, et al. 2024. 'A Global Meta-Analysis of Ecological Effects from Offshore Marine Artificial Structures'. *Nature Sustainability*. https://doi.org/10.1038/s41893-024-01311-z.

Leontiou, AJ, W Wu, and NJ Brown-Peterson. 2021a. 'Immature and Mature Female Red Snapper Habitat Use in the North-Central Gulf of Mexico'. *REGIONAL STUDIES IN MARINE SCIENCE* 44 (May). https://doi.org/10.1016/j.rsma.2021.101715.

Leontiou, AJ, W Wu, and NJ Brown-Peterson. 2021b. 'The Role of Maturity in Artificial Habitat Selection by Female Red Snapper'. *MARINE AND COASTAL FISHERIES* 13 (4): 332–44. https://doi.org/10.1002/mcf2.10160.

Løkkeborg, Svein, Odd-Børre Humborstad, Terje Jørgensen, and Aud Vold Soldal. 2002. 'Spatio-Temporal Variations in Gillnet Catch Rates in the Vicinity of North Sea Oil Platforms.' *ICES Journal of Marine Science/Journal Du Conseil* 59. https://dabred.imr.no/publications/sendFile/485/Lokkeborg_et_al_2002.pdf.

Madgett, AS, ES Harvey, D Driessen, KD Schramm, LAF Fullwood, S Songploy, J Kettratad, et al. 2022. 'Spawning Aggregation of Bigeye Trevally, Caranx Sexfasciatus, Highlights the Ecological Importance of Oil and Gas Platforms'. *ESTUARINE COASTAL AND SHELF SCIENCE* 276 (October). https://doi.org/10.1016/j.ecss.2022.108024.

Marnane, MJ, KD Schramm, D Driessen, LA Fullwood, BJ Saunders, S Songploy, J Kettratad, et al. 2022. 'Evidence of Fish Following Towed Oil and Gas Platforms to a Reefing Site and Rapid Colonisation'. *MARINE ENVIRONMENTAL RESEARCH* 180 (September). https://doi.org/10.1016/j.marenvres.2022.105728.

Montagna, Paul A, Stephen C Jarvis, and Mahlon C Kennicutt, Ii. 2002. 'Distinguishing between Contaminant and Reef Effects on Meiofauna near Offshore Hydrocarbon Platforms in the Gulf of Mexico'. *Canadian Journal of Fisheries and Aquatic Sciences* 59 (10): 1584–92. https://doi.org/10.1139/f02-131.

Mugge, RL, CF Rakocinski, M Woolsey, and LJ Hamdan. 2023. 'Proximity to Built Structures on the Seabed Promotes Biofilm Development and Diversity'. *BIOFOULING* 39 (7): 706–18. https://doi.org/10.1080/08927014.2023.2255141.

Plumlee, J.D., D.N. Hala, J.R. Rooker, J.B. Shipley, and R.J.D. Wells. 2021. 'Trophic Ecology of Fishes Associated with Artificial Reefs Assessed Using Multiple Biomarkers'. *Hydrobiologia* 848 (18): 4347–62. https://doi.org/10.1007/s10750-021-04647-1.

Plumlee, Jeffrey D., Kaylan M. Dance, Michael A. Dance, Jay R. Rooker, Thomas C. TinHan, J. Brooke Shipley, and R. J. Wells. 2020. 'Fish Assemblages Associated with Artificial Reefs Assessed Using Multiple Gear Types in the Northwest Gulf of Mexico'. *Bulletin of Marine Science* 96 (4): 655–78.

Ponti, Massimo, Marco Abbiati, and Victor Ugo Ceccherelli. 2002. 'Drilling Platforms as Artificial Reefs: Distribution of Macrobenthic Assemblages of the "Paguro" Wreck (Northern Adriatic Sea)'. *ICES Journal of Marine Science* 59 (suppl): S316–23.

Reynolds, Emily M., James H. Cowan Jr, Kristy A. Lewis, and Kirsten A. Simonsen. 2018. 'Method for Estimating Relative Abundance and Species Composition around Oil and Gas Platforms in the Northern Gulf of Mexico, USA'. *Fisheries Research* 201:44–55.

Rezek, Ryan J., Benoit Lebreton, Terence A. Palmer, Gregory W. Stunz, and Jennifer Beseres Pollack. 2018. 'Structural and Functional Similarity of Epibenthic Communities on Standing and Reefed Platforms in the Northwestern Gulf of Mexico'. *Progress in Oceanography* 168:145–54.

Sammarco, P. W., A. Lirette, Y. F. Tung, G. S. Boland, M. Genazzio, and J. Sinclair. 2014. 'Coral Communities on Artificial Reefs in the Gulf of Mexico: Standing vs. Toppled Oil Platforms'. *ICES Journal of Marine Science* 71 (2): 417–26.

Sammarco, Paul W., Scott A. Porter, and Stephen D. Cairns. 2010. 'A New Coral Species Introduced into the Atlantic Ocean Tubastraea Micranthus (Ehrenberg 1834)(Cnidaria, Anthozoa, Scleractinia): An Invasive Threat?' *Aquatic Invasions*. https://repository.si.edu/bitstream/handle/10088/10288/iz_2010_Invasive.pdf.

Schwartzkopf, Brittany D., Todd A. Langland, and James H. Cowan Jr. 2017. 'Habitat Selection Important for Red Snapper Feeding Ecology in the Northwestern Gulf of Mexico'. *Marine and Coastal Fisheries* 9 (1): 373–87.

Seaman Jr, William, William J. Lindberg, Carter R. Gilbert, and Thomas K. Frazer. 1989. 'Fish Habitat Provided by Obsolete Petroleum Platforms off Southern Florida'. *Bulletin of Marine Science* 44 (2): 1014–22.

Sibley, ECP, AS Madgett, TS Elsdon, MJ Marnane, ES Harvey, S Songploy, J Kettradad, and PG Fernandes. 2023. 'An Acoustic-Optic Comparison of Fish Assemblages at a Rigs-to-Reefs Habitat and Coral Reef in the Gulf of Thailand'. *ESTUARINE COASTAL AND SHELF SCIENCE* 295 (December). https://doi.org/10.1016/j.ecss.2023.108552.

Simonsen, Kirsten A., James H. Cowan, and Kevin M. Boswell. 2015. 'Habitat Differences in the Feeding Ecology of Red Snapper (Lutjanus Campechanus, Poey 1860): A Comparison between Artificial and Natural Reefs in the Northern Gulf of Mexico'. *Environmental Biology of Fishes* 98 (3): 811–24. https://doi.org/10.1007/s10641-014-0317-9.

Soldal, Aud Vold, Ingvald Svellingen, Terje Jørgensen, and Svein Løkkeborg. 2002. 'Rigs-to-Reefs in the North Sea: Hydroacoustic Quantification of Fish in the Vicinity of a "Semi-Cold" Platform'. *ICES Journal of Marine Science* 59 (suppl): S281–87.

Streich, Matthew K., Matthew J. Ajemian, Jennifer J. Wetz, and Gregory W. Stunz. 2017. 'A Comparison of Fish Community Structure at Mesophotic Artificial Reefs and Natural Banks in the Western Gulf of Mexico'. *Marine and Coastal Fisheries* 9 (1): 170–89.

Streich, Matthew K., Matthew J. Ajemian, Jennifer J. Wetz, Jason A. Williams, J. Brooke Shipley, and Gregory W. Stunz. 2017. 'A Comparison of Size Structure, Age, and Growth of Red Snapper from Artificial and Natural Habitats in the Western Gulf of Mexico'. *Transactions of the American Fisheries Society* 146 (4): 762–77.

Szostek, C. L., S. C. L. Watson, N. Trifonova, N. J. Beaumont, and B. E. Scott. 2025. 'Spatial Conflict in

Wanless, Ross M., Sue Scott, Warwick H. H. Sauer, Timothy G. Andrew, James P. Glass, Brian Godfrey, Charles Griffiths, and Eleanor Yeld. 2010. 'Semi-Submersible Rigs: A Vector Transporting Entire Marine Communities around the World'. *Biological Invasions* 12 (8): 2573–83. https://doi.org/10.1007/s10530-009-9666-2.
